# Key role of K^+^ and Ca^2+^ in high-yield ethanol production by *S. Cerevisiae* from concentrated sugarcane molasses

**DOI:** 10.1186/s12934-024-02401-5

**Published:** 2024-05-09

**Authors:** Wei-Yang Wang, Bei-Ping Wang, Hai-Song Su, Mei-Ming Wei, Yu-Tuo Wei, Fu-Xing Niu

**Affiliations:** 1 Guangxi Key Laboratory of Green Processing of Sugar Resources, Guangxi University of Science and Technology, Liuzhou, 545006 China; 2 Guangxi Microorganism and Enzyme Research Center of Engineering Technology, College of Life Science and Technology, Guangxi University, Nanning, 530004 Guangxi China

**Keywords:** *Saccharomyces cerevisiae*, Sugarcane molasses, Ethanol synthesis, Omics analysis

## Abstract

**Background:**

*Saccharomyces cerevisiae* is an important microorganism in ethanol synthesis, and with sugarcane molasses as the feedstock, ethanol is being synthesized sustainably to meet growing demands. However, high-concentration ethanol fermentation based on high-concentration sugarcane molasses—which is needed for reduced energy consumption of ethanol distillation at industrial scale—is yet to be achieved.

**Results:**

In the present study, to identify the main limiting factors of this process, adaptive laboratory evolution and high-throughput screening (Py-Fe^3+^) based on ARTP (atmospheric and room-temperature plasma) mutagenesis were applied. We identified high osmotic pressure, high temperature, high alcohol levels, and high concentrations of K^+^, Ca^2+^, K^+^ and Ca^2+^ (K^+^&Ca^2+^), and sugarcane molasses as the main limiting factors. The robust *S. cerevisiae* strains of NGT-F1, NGW-F1, NGC-F1, NGK^+^, NGCa^2+^ NGK^+^&Ca^2+^-F1, and NGTM-F1 exhibited high tolerance to the respective limiting factor and exhibited increased yield. Subsequently, ethanol synthesis, cell morphology, comparative genomics, and gene ontology (GO) enrichment analysis were performed in a molasses broth containing 250 g/L total fermentable sugars (TFS). Additionally, *S. cerevisiae* NGTM-F1 was used with 250 g/L (TFS) sugarcane molasses to synthesize ethanol in a 5-L fermenter, giving a yield of 111.65 g/L, the conversion of sugar to alcohol reached 95.53%. It is the highest level of physical mutagenesis yield at present.

**Conclusion:**

Our results showed that K^+^ and Ca^2+^ ions primarily limited the efficient production of ethanol. Then, subsequent comparative transcriptomic GO and pathway analyses showed that the co-presence of K^+^ and Ca^2+^ exerted the most prominent limitation on efficient ethanol production. The results of this study might prove useful by promoting the development and utilization of green fuel bio-manufactured from molasses.

**Supplementary Information:**

The online version contains supplementary material available at 10.1186/s12934-024-02401-5.

## Background

Energy shortage and food security are of great concern globally, and although fossil fuels offer huge short-term benefits, their long-term pollution will affect future generations. Therefore, developing green and sustainable biofuels that can viably replace fossil fuels is warranted. Recently, bioethanol has attracted much attention because it can achieve green biosynthesis. Apart from being a fuel with high octane number that can be mixed with existing energy substances, in this era of constant threats to human health, ethanol is also used widely as a medicine and food additive [1, 2]. Bioethanol is synthesized primarily via fermentation by *Saccharomyces cerevisiae* using various substrates such as glucose, sucrose, and starch. However, constant international turbulence means that food security also warrants attention, so synthesizing non-food bioethanol has become a key research direction.

**Fig. 1:**
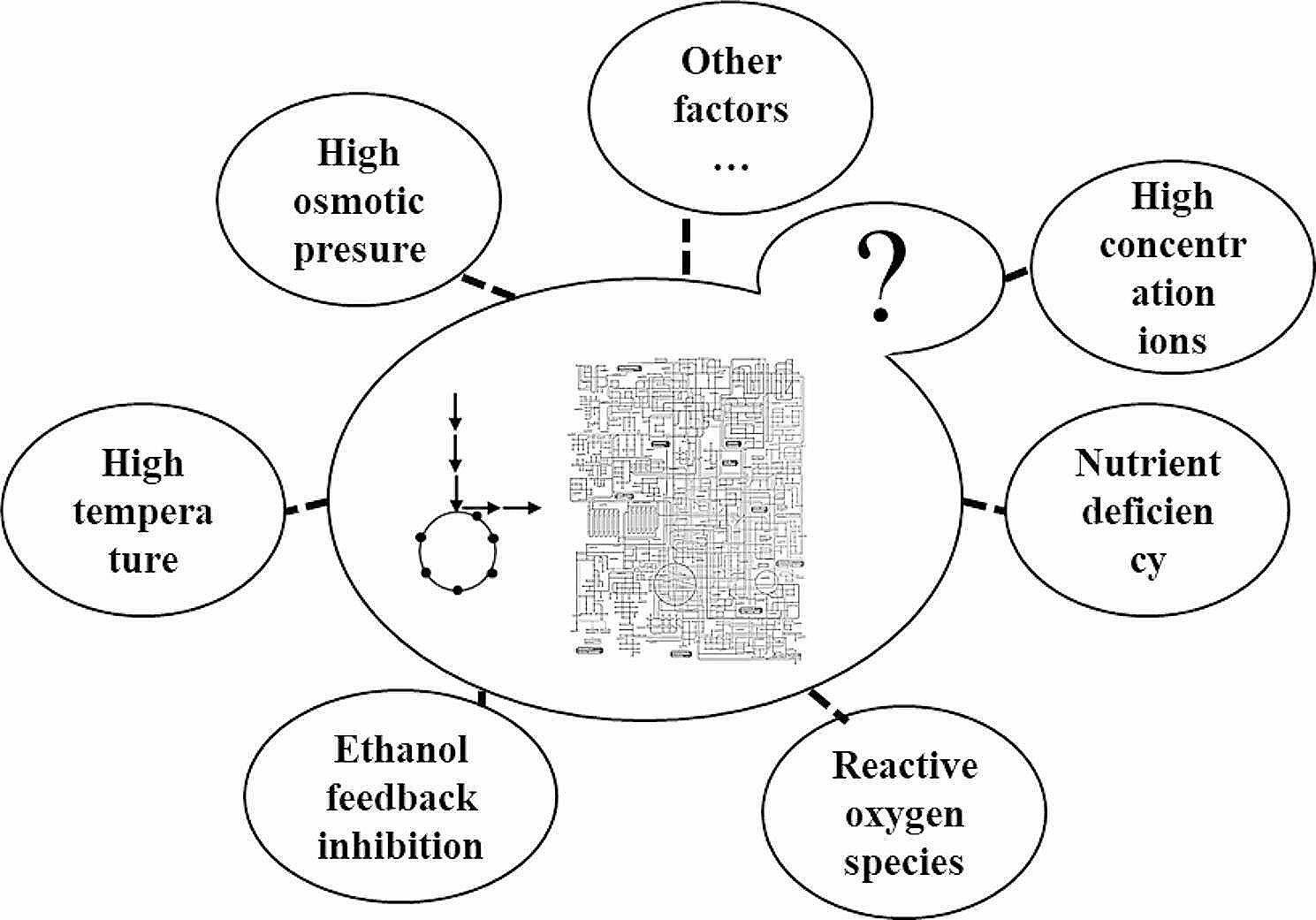
Factors limiting ethanol fermentation of *S. cerevisiae* in high concentration sugarcane molasses

Sugarcane molasses is a by-product of the sugar industry (three tons of sugar produce approximately one ton of molasses), containing 30–60% (w/v) sugar, colloids, and many metal ions [3–5]. Generally, molasses requires pretreatment before being exposed to *S. cerevisiae*. However, according to Abbott’s “alert level” theory [6], several other factors in molasses can restrict the growth and vitality of yeast cells, such as ethanol feedback inhibition, temperature, high osmotic pressure, and ion effects (Fig. 1). Different methods of fermentation [7, 8] and immobilization [9, 10] have been studied to overcome this issue, but the inherent genetic limitations of *S. cerevisiae* cells mean that it is still challenging to use high-concentration sugarcane molasses for ethanol fermentation. To that end, previous studies have explored and attempted to overcome various limiting factors (such as high osmotic pressure [11], high temperature [12–14], ethanol feedback inhibition [15], different ions [3], etc.) effectively ameliorate the restriction on *S. cerevisiae* growth and achieve ethanol fermentation from high-concentration sugarcane molasses [16, 17]. However, current research is in the bottleneck exploration stage, and research on the primary limiting factors is still either speculative or in the traditional stage [18]. Some of these factors do not apply to fermentation with sugarcane molasses as the substrate. For instance, there is a marked difference in ethanol yield after fermentation with sugarcane molasses or sucrose of the same total sugar concentration as the substrate, and this implies that ethanol feedback inhibition is not the main factor limiting ethanol synthesis from molasses fermentation [19, 20]. Similarly, the osmotic pressure formed by high sucrose concentration does not have a critical effect on *S. cerevisiae*. Previous studies have also proved that the stress factors in the culture of *S. cerevisiae* with low concentration of sugarcane molasses will still affect cell growth (data not shown), which is not conducive to biosynthesis based on sugarcane molasses as substrate. Therefore, identifying why *S. cerevisiae* struggles to use high-concentration molasses for high-concentration ethanol fermentation is crucial for future research.

Mutagenesis is an important means of tolerance engineering and is a highly promising technique for improving ethanol production of *S. cerevisiae*, especially under high-gravity fermentation conditions [21, 22]. In the study reported herein, we applied ARTP (atmospheric and room-temperature plasma) mutagenesis, ALE (adaptive laboratory evolution), and high-throughput screening (Py-Fe^3+^) [2] to obtain strains with robust performance against high sugar concentration (400 g/L total fermentable sugars (TFS)), high temperature (37℃), high alcohol level (10%(v/v)), and of 16 g/L K^+^, 8 g/L Ca^2+^, 16 g/L&8 g/L K^+^&Ca^2+^, and high sugarcane molasses (300 g/L TFS), and then ethanol production was performed in molasses broth containing 250 g/L TFS, and omics were studied. Herein, to the best of our knowledge and based on experimental verification, the major restricting factors of *S. cerevisiae* for high-concentration ethanol fermentation from sugarcane molasses are reported for the first time.

## Results

### Tolerance engineering improves ethanol production of *S. Cerevisiae*

It has been reported many times that bio-fuel synthesis can increase yields by increasing strain tolerance to factors that affect product synthesis [23–26]. Notably, the growth and vitality of *S. cerevisiae* cells are markedly restricted after exposure to high-concentration sugarcane molasses (Supplementary Fig. 1). when *S. cerevisiae* was cultured in molasses broth containing 250 g/L TFS for 24 h, a large proportion of yeast cells died, while the remaining cells were in a withered, thin, and adhesion state. The potential causal factors include high temperature; high ethanol levels; high sugar levels; and high concentrations of K^+^ and Ca^+^, the two most abundant ions (16 g/L and 8 g/L, respectively) in molasses. Therefore, to get more viable yeast cells for ethanol production, it is vital to improve the tolerance of yeast cells to the different restricting factors and obtain corresponding robust strains. Thus, *S. cerevisiae* GJ08 was subjected to ARTP mutagenesis and ALE under varying circumstances. Previously described [2] high-throughput approaches of triphenyl‑2 H‑tetrazoliumchloride (TTC)‑based macroscopic observation and the reaction of ferric nitrate with pyruvate (or pyruvate radical ion) in fermentation broth (Py-Fe^3+^) were used to screen the target strains (Table 1).

**Table 1:** Robust strains of *S. cerevisiae* selected in this study

Strain	characteristic	Production (g/L)	Ratio* (%)
*S. cerevisiae* NGT-F1	tolerance to 400 g/L TFS	142.02 ± 5.52	25.81
*S. cerevisiae* NGW-F1	tolerance to 37℃	119.97 ± 7.15	48.4
*S. cerevisiae* NGC-F1	tolerance to 10%(v/v) ethanol	119.83 ± 4.31	10.71
*S. cerevisiae* NGK^+^-F1	tolerance to 16 g/L K^+^	92.27 ± 3.01	11.25
*S. cerevisiae* NGCa^2+^-F1	tolerance to 8 g/L Ca^2+^	92.41 ± 2.00	15.07
*S. cerevisiae* NGK^+^&Ca^2+^-F1	tolerance to 16 g/L K^+^ and 8 g/L Ca^2+^	85.13 ± 4.21	11.16
*S. cerevisiae* NGTM-F1	tolerance to 300 g/L (TFS) sugarcane molasses	102.30 ± 6.80	12.49

*Under the same fermentation conditions, compared with wild strain GJ08

High sugar concentration can cause increased osmotic pressure, affecting cell growth and ethanol production [27]. *S. cerevisiae* NGT-F1, tolerant to 400 g/L total sugar, was screened. This strain produced the highest level of ethanol (142.02 g/L) under 325 g/L TFS, which was 28.81% higher than the yield obtained from the original strain under similar conditions (Supplementary Figs. 2–7). *S. cerevisiae* NGT-F1 continues to be used for mutagenesis and screening of high-temperature tolerant strains.

Stress due to high temperature affects protein structures and function and gives rise to growth inhibition or cell death [28]. Ethanol synthesis by S. cerevisiae is carried out at 30 °C, during which cellular metabolism generates heat, as does the mechanical stirring of the fermentation equipment. Therefore, the actual temperature of industrial fermentation will be unstable (may float 3–5 degrees), which will affect the production of yeast cells. So, *S. cerevisiae* NGW-F1, tolerant to 37 °C, was screened. This strain produced the highest level of ethanol (119.97 g/L) under 400 g/L TFS, which was 48.4% higher than the yield obtained from the original strain under similar conditions (Supplementary Figs. 8–12).

Stress due to high ethanol concentration affects cellular wall permeability, disrupting sorting and signaling functions [29]. *S. cerevisiae* NGC-F1, tolerant to 10% (v/v) ethanol, was screened. This strain produced the highest level of ethanol (119.83 g/L) under 250 g/L TFS, which was 10.71% higher than the yield obtained from the original strain under similar conditions (Supplementary Figs. 13–17).

K^+^ and Ca^2+^ are the largest components among the many ions in molasses. Another strain *S. cerevisiae* NGK^+^&Ca^2+^-F1, tolerant to 16 g/L K^+^ and 8 g/L Ca^2+^, was screened. This strain produced the highest level of ethanol (85.13 g/L) under 200 g/L TFS, which was 11.16% higher than the yield obtained from the original strain under similar conditions (Supplementary Figs. 30–35).

Finally, *S. cerevisiae* NGTM-F1, tolerant to 300 g/L TFS of sugarcane molasses, was screened. This strain produced the highest level of ethanol (102.30 g/L) under 250 g/L TFS, which was 12.49% higher than the yield obtained from the original strain under similar conditions (Supplementary Figs. 36–41). As shown in Supplementary Fig. 7A and B, 12 A and B, 35 A and B, and 41 A and B, compared with the control strains, the tolerant strains exhibited greatly improved cell morphology and were rounded and non-sticky.

### Stirred-tank fermenter fermentation for ethanol production

**Fig. 2:**
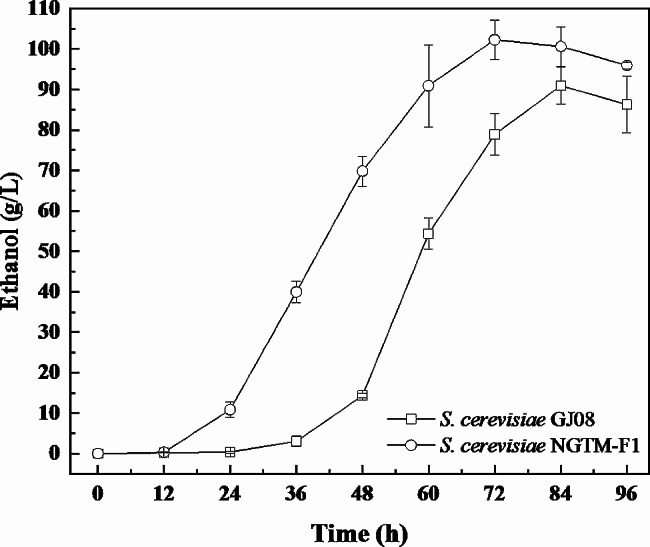
Ethanol synthesis curve over time. 30 ℃, sugarcane molasses containing 250 g/L total fermentable sugars (TFS), 10% inoculum □: *S. cerevisiae* GJ08; ○: *S. cerevisiae* NGTM-F1

The robust *S. cerevisiae* NGTM-F1 strain completely broke the restriction from high-concentration sugarcane molasses, making it easy to reveal the major limiting factors of high-concentration ethanol fermentation from high-concentration sugarcane molasses. First, the dynamic of ethanol biosynthesis was analyzed (Fig. 2). Results show that under similar conditions (30 °C, 250 g/L TFS, 10% inoculum), compared with the GJ08 strain, *S. cerevisiae* NGTM-F1 exhibited higher ethanol synthesis capacity and shorter optimal duration of ethanol synthesis (less by 12 h). This finding might be attributed to improved tolerance and growth of the engineered strain.

**Fig. 3:**
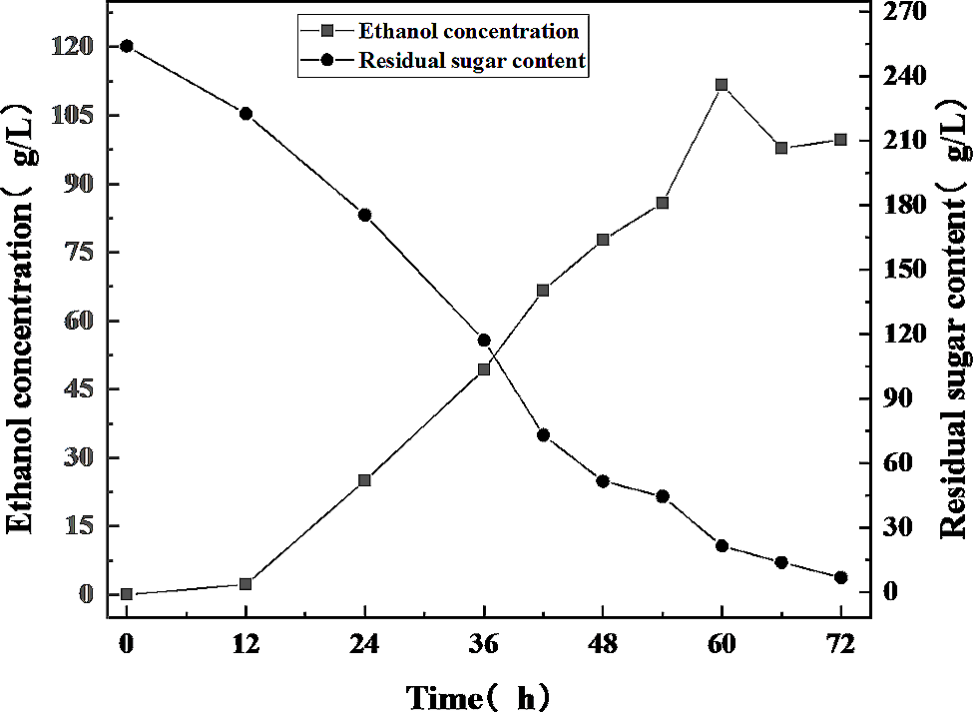
Stirred-tank fermenter fermentation for ethanol production. 250 g/L (TFS) sugarcane molasses, 30℃, 150 rpm, 17.5% inoculum, 2.2 g/L urea, micro-oxygenation (80 mL/min)

Furthermore, a 5-L fermenter was used to demonstrate the ethanol synthesis performance of *S. cerevisiae* NGTM-F1. The amounts of inoculum and urea added into the fermenter were optimized (17.5% and 2.2 g/L, respectively; Supplementary Figs. 42 and 43). The microaeration strategy was used for the fermentation process. Though *S. cerevisiae*-mediated ethanol fermentation occurs in an anaerobic environment, oxygen is still required for yeast cell growth, and a limited amount of oxygen can lead to better ethanol production [30]. Previous studies have shown that micro-oxygenation is the most efficient fermentation approach (80 mL/min, details not shown). In the current study, at 60 h, the ethanol yield reached a maximum of 111.65 g/L (14.15%(v/v)). With a 21.34 g/L residual sugar and calculated with Eq. 1, a sugar conversion rate of 95.53%, and a production rate of 1.86 g/L/h were obtained (Fig. 3). Wu et al. (2020) enhanced ethanol production from sugarcane molasses by using the engineered *S. cerevisiae* strain (*PHO4* gene replaced), which is a fast-growing strain, and achieved the highest production (114.71 g/L) at 56 h [31]. In the present study, physical mutagenesis (not gene editing) was used to achieve similar results. To date, 111.65 g/L ethanol a sugar conversion rate of 95.53%, and a production rate of 1.86 g/L/h (in this paper) is the highest yield obtained from high-concentration sugarcane molasses using an *S. cerevisiae* strain obtained from physical mutagenesis. Physical mutagenesis is superior to gene editing in building robust strains. Gene editing is mostly limited to the major known genes, and the number of genes that can be edited at the same time is limited. Physical mutagenesis cannot be limited by the type and number of genes, and the operation is simple. This strain is now stored at Guangdong Microbial Culture Collection Center (GDMCC 63,687).

### Fermentation with tolerant strains using high-concentration sugarcane molasses

**Fig. 4:**
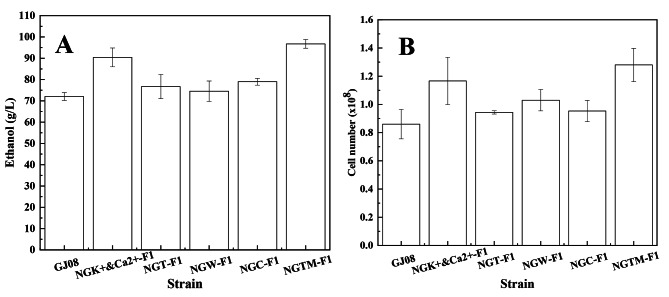
Ethanol synthesis yield and cell number of *S. cerevisiae* GJ08, NGK^+^&Ca^2+^-F1, NGT-F1, NGW-F1, NGC-F1, and NGTM-F1 in 250 g/L (TFS) sugarcane molasses. **A**: Ethanol synthesis yield of different *S. cerevisiae*; **B**: Number of different *S. cerevisiae* cells

In this study, *S. cerevisiae* NGTM-F1 was found to counter the effects of high-concentration sugarcane molasses, and it can be used as a good reference to identify the main limiting factors in the fermentation of molasses. In theory, countering the primary limiting factors will improve the fermentation efficiency of high-concentration sugarcane molasses. Thus, *S. cerevisiae* NGT-F1, NGC-F1, NGW-F1, NGK^+^&Ca^2+^-F1, and NGTM-F1 were separately subjected to ethanol fermentation under the same conditions (sugarcane molasses containing 250 g/L TFS) (Fig. 4A). As shown in Fig. 4A, S. *cerevisiae* NGT-F1, NGC-F1, NGW-F1, and NGK^+^&Ca^2+^-F1 provided improved ethanol yields compared to that with the original strain *S. cerevisiae* GJ08. This indicates that improving the robustness of *S. cerevisiae* can promote the biosynthesis process using sugarcane molasses as a substrate. However, *S. cerevisiae* NGTM-F1 gave the highest ethanol yield of all the engineered strains, followed closely by only *S. cerevisiae* NGK^+^&Ca^2+^-F1. Moreover, the number of cells showed the same trend as ethanol production (Fig. 4B). Thus, the influence of ions on sugarcane molasses was shown to be more important.

**Fig. 5:**
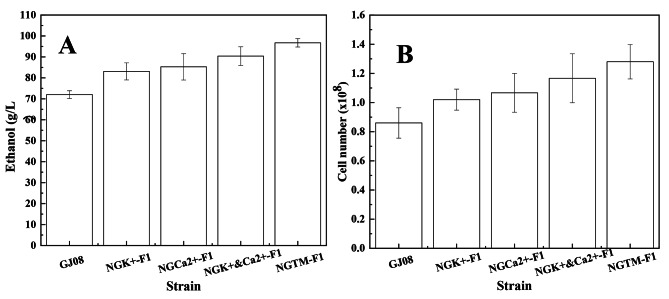
Ethanol synthesis yield and cell number of *S. cerevisiae* GJ08, *S. cerevisiae* NGK^+^-F1, NGCa^2+^-F1, NGK^+^&Ca^2+^-F1, and NGTM-F1 in 250 g/L (TFS) sugarcane molasses. **A**: Ethanol synthesis yield of different *S. cerevisiae*; **B**: Number of different *S. cerevisiae* cells

**Fig. 6:**
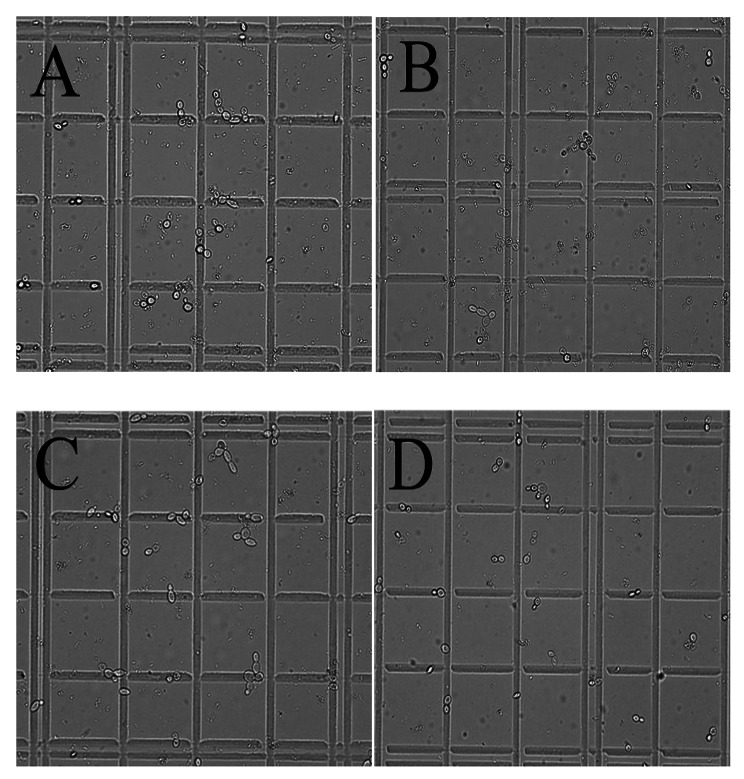
Cell morphology of different *S. cerevisiae* cultured in 250 g/L molasses. **A**: *S. cerevisiae* NGCa^2+^-F1; **B**: *S. cerevisiae* NGK^+^-F1; **C**: *S. cerevisiae* NGK^+^&Ca^2+^-F1; **D**: *S. cerevisiae* NGTM-F1

Chotineeranat et al. (2010) reported that Ca^2+^ inhibited *S. cerevisiae*-mediated fermentation using molasses as a substrate because of the inhibitory effect of this ion on invertase, an enzyme that converts sucrose into invert sugars [3]. Tiligada et al. (2010) reported that the K^+^ channels open while the cell membrane is depolarized and involved in the transport of other compounds [32], and Merchan et al. (2011) reported that the accumulation of K^+^ renders yeast cells sensitive to DNA-damaging agents [33]. The composition of molasses is complex, and it is unknown whether the presence of potassium ions in large quantities will cause a chain reaction that damages cells. So, to assess the effects of K^+^ and Ca^2+^—either alone or together—on the fermentation of sugarcane molasses, *S. cerevisiae* NGK^+^-F1 and NGCa^2+^-F1 were constructed. We found that these strains gave a higher ethanol yield (Fig. 5A) and improved cell number (Fig. 5B) compared to the original strain. However, they did not reach the level of *S. cerevisiae* NGK^+^&Ca^2+^-F1. Moreover, the morphological maps of *S. cerevisiae* NGK^+^, NGCa^2+^, NGK^+^&Ca^2+^-F1, and NGTM-F1 cultured in 250 g/L molasses, were analyzed (Fig. 6A–D). As shown in Fig. 6, the cell morphology of *S. cerevisiae* NGK^+^&Ca^2+^-F1 and NGTM-F1 is similar, with larger cell size and fewer adherent cells. These results also indicate that the co-existence of K^+^ and Ca^2+^ in molasses is the key limiting factor for *S. cerevisiae*-mediated fermentation of high-concentration sugarcane molasses.

### Whole genome resequencing

Phylogenetic modeling based on genomes has been widely used in the field of virus development because it can obtain more accurate similarity [34]. Similarly, this method can also be applied to the comparison of microbial differences. To this end, the genomes of *S. cerevisiae* NGK^+^-F1, NGCa^2+^-F1, NGK^+^&Ca^2+^-F1, and NGTM-F1 were re-sequenced and compared. The whole genome was re-sequenced using a 2 × 150 paired-end configuration. Supplementary Table 1 shows the genome coverage and the ratio of clean bases after aligning to the genome sequence of *S. cerevisiae* S288C. The mutant bases in the engineered strains are shown in Supplementary Table 2.

**Fig. 7:**
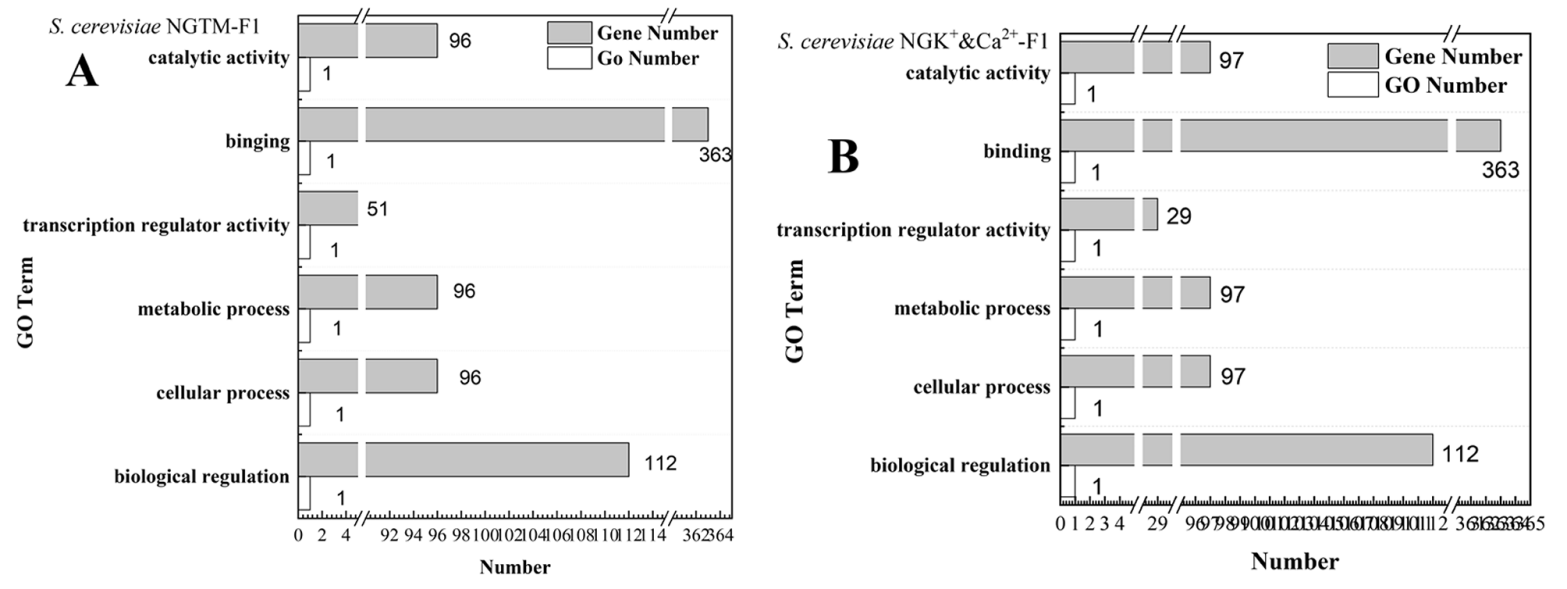
Whole genome resequencing GO enrich analysis. **A**: *S. cerevisiae* NGTM-F1; **B**: *S. cerevisiae* NGK^+^&Ca^2+^-F1

Next, we evaluated why the simultaneous presence of K^+^ and Ca^2+^ in sugarcane molasses inhibited the fermentation process more significantly than either ion alone, based on whole-genome resequencing. As shown in Supplementary Table 1, all coverages achieved more than 99%, but the number of mutations in *S. cerevisiae* NGK^+^-F1, NGCa^2+^-F1, and NGK^+^&Ca^2+^-F1 was not significantly different from that in *S. cerevisiae* NGTM-F1. Subsequently, the mutated genes (only the exon region) of the engineered strains were examined using gene ontology (GO) enrichment analysis (Fig. 7). GO enrichment analysis is mainly based on the selected differentially expressed genes using DAVID (https://david.ncifcrf.gov/) for gene ontology enrichment analysis, GO enrichment analysis mainly three Ontology to start, including molecular function, cellular components, and biological processes involved. Theoretically, the locus of mutation caused by the primary limiting factor should be significantly similar to that in *S. cerevisiae* NGTM-F1. As shown in Fig. 7A and B, the mutation region of *S. cerevisiae* NGK^+^&Ca^2+^-F1 was most similar to that of *S. cerevisiae* NGTM-F1, with the involved GO term regions (catalytic activity, binding, transcription regulator activity, metabolic process, cellular processes, and biological regulation) and the number of genes per region being close. However, the GO analysis results of other yeast strains were quite different (Supplementary Fig. 44).

### Comparative transcriptomic analysis

**Fig. 8:**
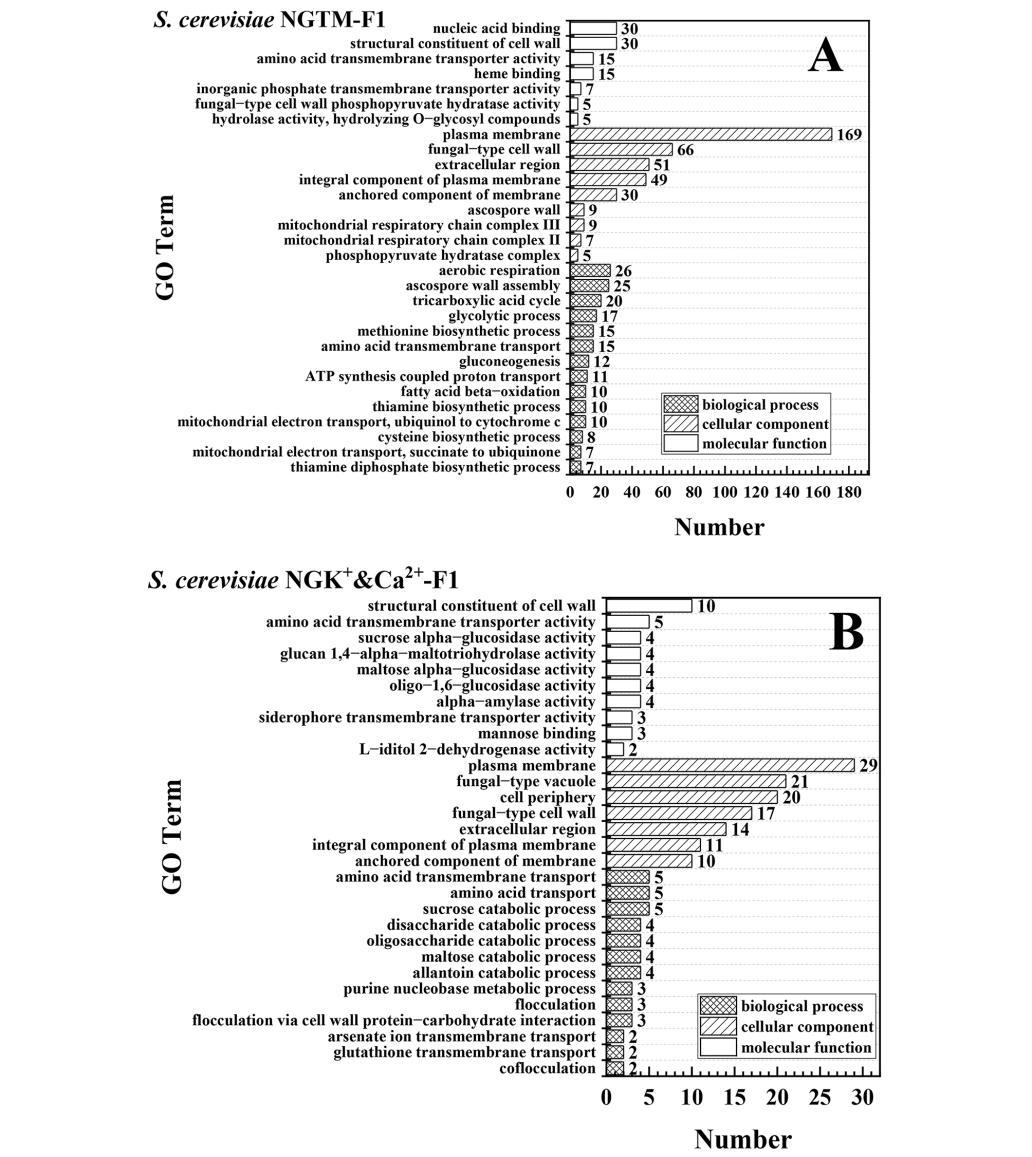
Comparative transcriptomic GO Term analysis. **A**: *S. cerevisiae* NGTM-F1 compared with *S. cerevisiae* GJ08 under 250 g/L (TFS) molasses; **B**: *S. cerevisiae* NGK^+^&Ca^2+^-F1 compared with *S. cerevisiae* GJ08 under 16 g/L K^+^ and 8 g/L Ca^2+^

Most phenotypic changes are accompanied by genotype changes. The transcription level changes induced by mutated genes can reflect the key mutations, and GO and Kyoto Encyclopedia of Genes and Genomes (KEGG) are better for comparing data differences between different groups. Therefore, *S. cerevisiae* NGK^+^-F1, NGCa^2+^-F1, NGK^+^&Ca^2+^-F1, and NGTM-F1 were analyzed via comparative transcriptomics against *S. cerevisiae* GJ08. To analyze the mutated genes more intuitively, GO analysis was used again. As shown in Fig. 8, the effects of the key limiting factors on *S. cerevisiae* cells were more biased to the cellular component. However, the overall bias distributions in *S. cerevisiae* NGTM-F1 and NGK^+^&Ca^2+^-F1were more similar than other robust strains (Supplementary Fig. 45). As shown in Fig. 8A and B, the plasma membrane was the most abundant cellular component, while the structural constituent of the cell wall was the primary component of the molecular function part. In addition, other parts such as amino acid transmembrane transporter activity, fungal-type vacuole, extracellular region, and amino acid transmembrane transport also accounted for a considerable proportion.

**Fig. 9:**
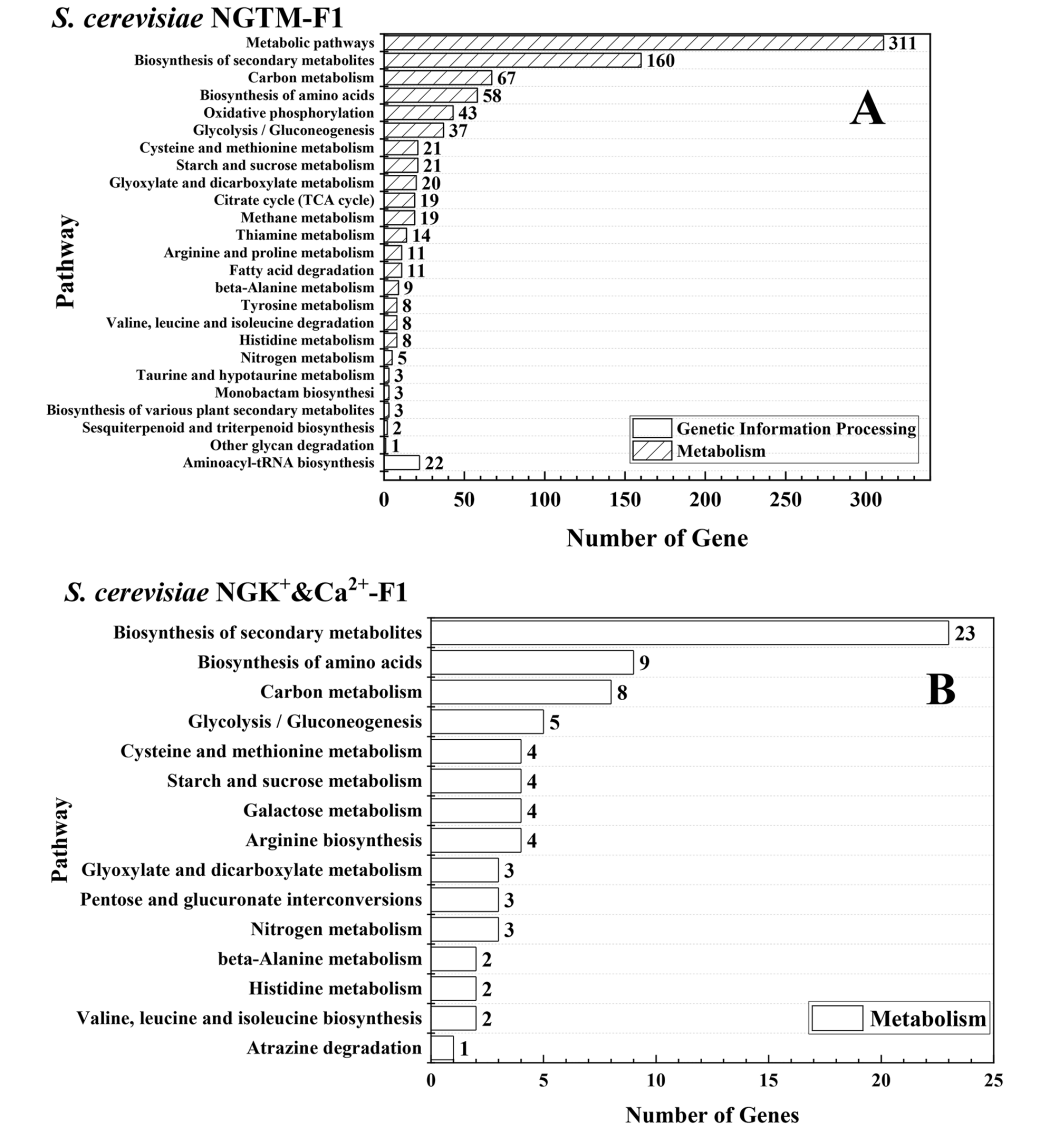
Analysis and comparison of *S. cerevisiae* NGK^+^&Ca^2+^-F1 and *S. cerevisiae* NGTM-F1 Pathway. **A**: *S. cerevisiae* NGTM-F1; **B**: *S. cerevisiae* NGK^+^&Ca^2+^-F1

Next, KEGG was used to further analyze (Fig. 9) and compare the major regional genes (Supplement KEGG-gene). Furthermore, as shown in Table 2, the proportions of the affected genes of *S. cerevisiae* NGK^+^&Ca^2+^-F1 involved in the biosynthesis of secondary metabolites, biosynthesis of amino acids, carbon metabolism, glycolysis/gluconeogenesis, cysteine and methionine metabolism, and starch and sucrose metabolism were 91.3%, 100%, 87.5%, 100%, 100%, and 75%, respectively, which were identical to those of the affected genes of *S. cerevisiae* NGTM-F1. In particular, it should be noted that these overlapping genes showed a high degree of consistency in the process of transcriptional changes, that is, genes that were up-regulated in *S. cerevisiae* NGK^+^&Ca^2+^-F1 were also up-regulated in *S. cerevisiae* NGTM-F1. Genes that are down-regulated in *S. cerevisiae* NGK^+^&Ca^2+^-F1 are also down-regulated in *S. cerevisiae* NGTM-F1.

**Table 2:** Changes in transcription levels of *S. cerevisiae* NGK^+^&Ca^2+^-F1vs *S. cerevisiae* NGTM-F1 in major region

path-way Factor	Biosynthesis of secondary metabolites		Biosynthesis of amino acids		Carbon metabolism		Glycolysis / Gluconeogenesis		Cysteine and methionine metabolism		Starch and sucrose metabolism	
	N	O	N	O	N	O	N	O	N	O	N	O
Cane molasses	160	21	58	9	67	7	37	5	21	4	21	3
K^+^&Ca^2+^	23		9		8		5		4		4	

N: The number of mutated genes whose transcription levels have changed

O: The number of identical genes was compared between the mutated genes with changes in pathway transcription level caused by K^+^&Ca^2+^ and those with changes in pathway transcription level sent by sugarcane molasses

*The data contain both increases and decreases in transcripts (the transcriptional level changes of genes were the same), and the screening criteria for significant enrichment was Q value < = 0.05

Sugarcane molasses is a mixture that comprises several limiting factors, but the finding of K^+^ and Ca^2+^ being the key limiting factors is a major breakthrough in the ethanol fermentation of high-concentration sugarcane molasses. This finding is of great significance for the development and utilization of green biomanufacturing with molasses as a substrate.

## Conclusions

In summary, we developed *S. cerevisiae* tolerant to high temperature, high osmotic pressure, high alcohol levels, and high concentrations of K^+^, Ca^2+^, K^+^&Ca^2+^, and sugarcane molasses, i.e., *S. cerevisiae* NGT-F1, NGC-F1, NGW-F1, NGK^+^-F1, NGCa^2+^-F1, NGK^+^&Ca^2+^-F1, and NGTM-F1, respectively. Our results showed that the co-existence of K^+^ and Ca^2+^ was the main limiting factor of *S. cerevisiae*-mediated ethanol fermentation of high-concentration sugarcane molasses. With 250 g/L (TFS) of sugarcane molasses, *S. cerevisiae* NGTM-F1 gave an ethanol yield, sugar conversion rate, and production rate of 111.65 g/L, 95.53%, and 1.86 g/L/h, respectively. These values were the highest reported by any study on *S. cerevisiae* subjected to physical mutation. Of course, after solving the problem of the main limiting factor of molasses, *S. cerevisiae* may be able to biosynthesize at higher molasses concentrations. Future studies must focus on using omics analysis to elucidate the mechanisms underlying simultaneous K^+^ and Ca^2+^ stress on *S. cerevisiae*-mediated ethanol fermentation. The results of this study have important guiding significance for not only the synthesis of ethanol by *S. cerevisiae* using molasses but also the subsequent synthesis of other substances using molasses as a substrate.

## Materials and methods

### Strains

The *S. cerevisiae* strains used in this study are list in Table 3.

**Table 3:** Strains used in this study

Strain	Description	Source
*S. cerevisiae* S288C	MATα; SUC2; gal2; mal2; mel; flo1; flo8-1; hap1; bio1; bio6	ATCC 204,508
*S. cerevisiae* GJ08	Mutant strain, derived from *S. cerevisiae* S288C	This study
*S. cerevisiae* NGT-F1	The ALE strain from *S. cerevisiae* GJ08, tolerance to 400 g/L total fermentable sugars (TFS)	[2]
*S. cerevisiae* NGC-F1	The ALE strain from *S. cerevisiae* GJ08, tolerance to 10% (v/v) ethanol	This study
*S. cerevisiae* NGW-F1	The ALE strain from *S. cerevisiae* GJ08, tolerance to 37℃	This study
*S. cerevisiae* NGK^+^-F1	The ALE strain from *S. cerevisiae* GJ08, tolerance to 16 g/L K^+^	This study
*S. cerevisiae* NGCa^2+^-F1	The ALE strain from *S. cerevisiae* GJ08, tolerance to 8 g/L Ca^2+^	This study
*S. cerevisiae* NGK^+^&Ca^2+^-F1	The ALE strain from *S. cerevisiae* GJ08, tolerance to 16 g/L K^+^ and 8 g/L Ca^2+^	This study
*S. cerevisiae* NGTM-F1	The ALE strain from *S. cerevisiae* GJ08, tolerance to 300 g/L (TFS) sugarcane molasses	This study, stored at Guangdong Microbial Culture Collection Center (GDMCC 63,687)

### Pretreatment of sugarcane molasses

Cane molasses was obtained from the Guangxi Sugar Industry Group (Guangxi, China), containing 12% (w/w) converted sugars (fructose and glucose), 35% (w/w) sucrose, 2.5% (w/w) other carbohydrates, 9.6% (w/w) ash, 4.3% (w/w) crude protein, 0.06% (w/w) crude fat, and various ions (K^+^ at 16 g/L, Ca^2+^ at 8 g/L, Mg^2+^ at 2.7 g/L, Fe^3+^ at 0.35 g/L, Mn^2+^ at 0.03 g/L, P^3+^ at 0.03 g/L, and other ions in smaller quantities). The molasses pretreatment process was as follows. The molasses was diluted with distilled water (1:1) and then boiled for 30 min while being stirred constantly. After cooling to room temperature, the pH of the mixture was adjusted to 1–2 with concentrated sulfuric acid. After being left overnight, 1% activated carbon powder was added to the molasses treatment solution at 90 °C for 30 min. After cooling to room temperature again, the supernatant was centrifuged at 4000 rpm for 10 min, and its pH was adjusted to 4.5–5.0 with calcium hydroxide. The supernatant was then centrifuged again at 4000 rpm for 10 min and then collected for testing.

### ARTP-based random mutagenesis and adoptive evolution

A 5-mL cell broth cultured with YPD medium (yeast extract 10 g/L, peptone 20 g/L, dextrose 20 g/L) for ca. 12 h was harvested by centrifugation and washed twice with ice-cold water. Then, ARTP mutagenesis was carried out using an ARTP mutation system (ARTP-C2-5; Tmaxtree Biotechnology Co., Ltd., Wuxi, China) as described by Niu et al. [35]. The parameters were set as follows: the radio-frequency power input was 100 W, the flow of helium was 10 SLM, the distance between the plasma torch nozzle exit and the slide was 2 mm, and the treatment time was selected as being 200, 180, 160, 140, 120, 100, or 80 (s). After treatment, the slides were washed with ice-cold water to generate the ARTP mutant library, and the mutant strains were transferred separately to the following different environments: (i) YPD medium with 286 g/L sucrose (ca. 300 g/L total sugar); (ii) YPD medium with 10% (v/v) ethanol; (iii) YPD medium with 8 g/L KCl; (iv) YPD medium with 4 g/L CaCl_2_; (v) YPD medium with 8 g/L KCl and 4 g/L CaCl_2_; (vi) YPD medium at 35 °C; (vii) 250 g/L TFS sugarcane molasses. Every 24 h, the number of cells was counted using a blood counting plate until the number stabilized, then ARTP mutagenesis was performed again, and the passage was gradually increased to higher gradient.

### TTC and Py-Fe3^+^-based screening

The library of ARTP-mutagenesis mutations of *S. cerevisiae* that could tolerate the specific environments was diluted and then coated on a YPD solid medium. According to our previous research results, the yeast with active cell growth has a stronger ethanol metabolism capacity, and its ethanol production is inversely proportional to the content of the precursor pyruvate. Therefore, TTC (triphenyl-2 H-tetrazoliumchloride) and Py-Fe3^+^-based screening was carried as described in previous work [2]. So, after 24 h of cultivation, 20 mL of TTC solution was introduced to react for 5 min, and the yeast strains with the earliest red and darkest color were selected. Next, single *S. cerevisiae* colonies on the slant plates were transferred to 48-deep-well microtiter plates (DWMPs) containing 1 mL of corresponding environmental liquid medium for incubation (described in Sect. 2.3). After fermentation, the DWMPs were left to rest for 30 min to allow the *S. cerevisiae* strains to settle automatically. Then 120 µL of the fermentation supernatant (five times dilution with ultrapure water) was transferred to a 96-well enzyme label plate, and 80 µL of 0.1 M Fe(NO_3_)_3_ was added for reaction at room temperature for 10 min. Then the absorbance was measured at 520 nm. Finally, several strains with low A_520_ values were selected for subsequent shake-flask fermentation and re-screening.

### Shake-flash fermentation

Single colonies of the respective mutant yeast strains were picked into 50 ml YPD medium for overnight incubation. The seed solution was then transferred to 250 ml triangular flasks containing 100 ml of the corresponding environment-specific medium at 10% inoculum and wrapped in breathable film and kraft paper to prevent ethanol volatilization.

### Fermentation in a stirred tank

The seed cultures were prepared by inoculating a single colony in a 250 mL Erlenmeyer flask containing 50 mL of YPD medium and then incubating for 12–14 h at 30 °C. The culture solution was collected by centrifugation, then sterile water was added to prepare 10-fold concentrated seed solution, and inoculated into 50 mL YPD medium (yeast extract 10 g/L, peptone 20 g/L, dextrose 50 g/L) at 10% inoculum for secondary seed culture for 8–10 h. Finally, it was inoculated at 17.5% into a 5-L fermenter containing 250 g/L TFS, urea 2.2 g/L, peptone 1 g/L medium. The fermenter conditions were 30 °C, 150 rpm, oxygenated (at 80 mL/min) and samples were taken at 0, 12, 24, 36, 42, 48, 54, 60, 66, and 72 h. The sugar alcohol conversion rate is given by eq:

1 $$y=\frac{x\times 180}{(TFS-RS)\times 2\times 46}\times 100\%$$

Where y is the sugar alcohol conversion, x is the ethanol production, TFS is the total fermentable sugars, RS is the residual fermentable sugar.

### Whole genome resequencing

Genomic library construction and whole-genome resequencing were performed on the Illumina HiSeq/Nova 2 × 150 bp platform by Azenta (Suzhou, China). The paired-end reads were aligned to the reference genome of *S. cerevisiae* S288C. Potential mutations including point mutation and insertion/deletion were identified. To reduce the nuisance of unwanted genes, ClusterProfiler [36] was used to set *p*-values (*p* < 0.05), the Benjamini–Hochberg procedure [37] was used to perform multiple test correction, and the *Q* value was obtained (*Q* < 0.2).

### Transcriptome analysis

The total RNA of *S. cerevisiae* was collected and extracted from shaking-flask culture to the fastest growth stage (logarithmic phase). Different *S. cerevisiae* mutants were cultured under conditions that they could tolerate, and *S. cerevisiae* GJ08 as a control was also cultured under these conditions separately for the same time. Cells were collected by centrifuge and washed three times with 0.01 M PBS buffer solution, then 2–3 mL of Trizol cracking solution was added into the centrifuge tube and blown evenly with a pipette gun. After cracking for 2–3 min, the yeast were immediately frozen in liquid nitrogen and stored in a refrigerator at − 80 °C. Transcriptome sequencing was performed on the Illumina/MGI platform by Azenta (Suzhou, China). The quality of the sequencing data was evaluated by FastQC (v0.10.1), and the error rate of sequencing was less than 0.5% for each base position.

### Assay

The number of cells was counted using a hemocytometer, and ethanol production was analyzed by GC-FID (Techcomp Scion 456-GC, Heolland). The inlet temperature was set to 200 °C, with the flow at 1 mL/min and the oven at 40 °C for 0 s, 40–80 °C for 5 min, and 80–150 °C for 10 min. The sugar content in the fermentation medium was determined by HPLC (RiLi, L2000), and the HPLC system consisted of an automatic injector equipped with an Alltima 5 μm amino column (250 × 4.6 mm). The mobile phase was 75% acetonitrile with a flow at 1 mL/min, and differential refraction detectors were used at 35 °C.

### Statistical analysis

All experiments were conducted in triplicate (Except fermentation in fermenters), and the data were averaged and presented in the form of mean value plus or minus standard deviation. One-way analysis of variance followed by Tukey’s test was used to determine significant differences using the OriginPro (version 9.1) package. Statistical significance was defined as *p* < 0.05.

### Electronic supplementary material

Below is the link to the electronic supplementary material.

Supplementary Material 1

Supplementary Material 2

## Data Availability

No datasets were generated or analysed during the current study.

## Abbreviations

ARTP: Atmospheric and Room-Temperature Plasma
ALE: Adaptive Laboratory Evolution
K^+^&Ca^2+^: K^+^ and Ca^2+^
GO: Gene Ontology
TFS: Total Fermentable Sugars
TTC: Triphenyl‑2 h‑Tetrazoliumchloride
DWMPs: Deep-Well Microtiter Plates
KEGG: Kyoto Encyclopedia of Genes and Genomes

## References

[1] Almeida IC, Pacheco TF, Machado F, Gonalves SB (2022). Evaluation of different strains of *Saccharomyces cerevisiae* for ethanol production from high-amylopectin BRS AG rice (Oryza sativaL). Sci Rep-UK.

[2] Wang WY, Wu SH, Xie YH, Zhong M, Wei ML, Li ZY (2022). A high-throughput screening procedure (Py-Fe^3+^) for enhancing ethanol production by *Saccharomyces cerevisiae* using ARTP random mutagenesis. Processes.

[3] Chotineeranat S, Wansuksri R, Piyachomkwan K, Chatakanonda P, Weerathaworn P, Sriroth K (2010). Effect of calcium ions on ethanol production from molasses by *Saccharomyces cerevisiae*. Sugar Tech.

[4] Ergun AT (2008). Effect of Zeolite NaY and Ca-montmorillonite on ethanol production using synthetic molasses. Appl Biochem Biotech.

[5] Kaseno, Kokugan T (1997). The effect of molasses pretreatment by ceramic microfiltration membrane on ethanol fermentation. J Ferment Bioeng.

[6] Abbott DA, Ingledew WM (2004). Buffering capacity of whole corn mash alters concentrations of organic acids required to inhibit growth of *Saccharomyces cerevisiae* and ethanol production. Biotechnol Lett.

[7] Echegaray OF, Carvalho J, Fernandes A, Sato S, Aquarone E, Vitolo M (2000). Fed-batch culture of *Sacchoromyces Cerevisiae* in sugar-cane blackstrap molasses: invertase activity of intact cells in ethanol fermentation. Biomass Bioenerg.

[8] Borzani W, Hiss H, Santos T, Vairo M (1992). Semicontinuous ethanol fermentation of sugar cane blackstrap molasses by pressed yeast. Biotechnol Lett.

[9] Ghorbani F, Younesi H, Sari A, Najafpour G (2011). Cane molasses fermentation for continuous ethanol production in an immobilized cells reactor by *Saccharomyces cerevisiae*. Renew Energ.

[10] Borovikova D, Scherbaka R, Patmalnieks A, Rapoport A (2014). Effects of yeast immobilization on bioethanol production. Appl Biochem.

[11] Perrier-Cornet JM, Hayert M, Saurat E, Milesse C, Gervais P. Effect of osmotic stress on high pressure inactivation of *Saccharomyces cerevisiae*. Conference Proceedings.1999;27–30.

[12] Andrade R, Rivera EC, Costa AC, Atala DIP, Filho RM (2007). Estimation of temperature dependent parameters of a batch alcoholic fermentation process. Appl Biochem Biotechnol.

[13] Khatun MM, Yu X, Kondo A, Bai F, Zhao X (2017). Improved ethanol production at high temperature by consolidated bioprocessing using *Saccharomyces cerevisiae* strain engineered with artificial zinc finger protein. Bioresource Technol.

[14] Baer S, Blaschek H, Smith T (1987). Effect of butanol challenge and temperature on lipid composition and membrane fluidity of butanol tolerant *Clostridium acetobutylicum*. Appl Environ Microb.

[15] Hoek JB, Rubin E (1990). Alcohol and membrane-associated signal transduction Alcohol. Alcohol Alcoholism.

[16] Qin L, Dong S, Yu J, Ning X, Li C (2020). Stress-driven dynamic regulation of multiple tolerance genes improves robustness and productive capacity of *Saccharomyces cerevisiae* in industrial lignocellulose fermentation. Metab Eng.

[17] Shima J, Takagi H (2009). Stress-tolerance of baker’s-yeast (*Saccharomyces cerevisiae*) cells: stress-protective molecules and genes involved in stress tolerance. Appl Biochem.

[18] Gibson BR, Lawrence SJ, Leclaire JPR, Powell CD, Smart KA (2010). Yeast responses to stresses associated with industrial brewery handling. FEMS Microbiol Rev.

[19] Junior MM, Batistote M, Cilli EM, Ernandes JR (2012). Sucrose fermentation by Brazilian ethanol production yeasts in media containing structurally complex nitrogen sources. J I Brew.

[20] Gasmalla M, Yang R, Nikoo M, Man S (2017). Production of ethanol from Sudanese sugar cane molasses and evaluation of its quality. J Food Process Technol.

[21] El-Hussieny NI, Bakri MM, Ganash M, Ghany TMA (2020). Chemical mutagenesis of *Saccharomyces cerevisiae* for enhancing bioethanol production with fermentation at very high sugar concentration. BioResources.

[22] Caspeta L, Castillo T, Nielsen J (2015). Modifying yeast tolerance to inhibitory conditions of ethanol production processes. Front Bioeng Biotechnol.

[23] Alper H, Moxley J, Nevoigt E, Fink GR, Stephanopoulos G (2006). Engineering yeast transcription machinery for improved ethanol tolerance and production. Science.

[24] Alsaker KV, Paredes C, Papoutsakis ET (2010). Metabolite stress and tolerance in the production of biofuels and chemicals: gene-expression-based systems analysis of butanol, butyrate, and acetate stresses in the anaerobe *Clostridium acetobutylicum*. Biotechnol Bioeng.

[25] Aono R (1998). Improvement of organic solvent tolerance level of *Escherichia coli* by overexpression of stress-responsive genes. Extremophiles.

[26] Niu FX, He X, Wu YQ, Liu JZ (2018). Enhancing production of pinene in *Escherichia coli* by using a combination of tolerance, evolution, and modular co-culture engineering. Front Microbiol.

[27] Stanley D, Bandara A, Fraser S, Chambers PJ, Stanley GA (2010). The ethanol stress response and ethanol tolerance of *Saccharomyces cerevisiae*. J Appl Microbiol.

[28] Goldberg AL (2003). Protein degradation and protection against misfolded or damaged proteins. Nature.

[29] Jones RP, Greenfield PF (1987). Ethanol and the fluidity of the yeast plasma membrane. Yeast.

[30] Alfenore S, Cameleyre X, Benbadis L, Bideaux C, Uribelarrea JL, Goma G (2004). Aeration strategy: a need for very high ethanol performance in *Saccharomyces cerevisiae* fed-batch process. Appl Microbiol Biot.

[31] Wu R, Chen D, Cao S, Lu Z, Huang J, Lu Q (2020). Enhanced ethanol production from sugarcane molasses by industrially engineered *Saccharomyces cerevisiae* via replacement of the *PHO4* gene. RSC Adv.

[32] Tiligada E, Delitheos A (2010). Involvement of potassium ions in the action of various antineoplastic drugs on the growth of *Saccharomyces cerevisiae*. Lett Appl Microbiol.

[33] Stephanie M, Leda, Pedelini, Guillem H (2010). Genetic alterations leading to increases in internal potassium concentrations are detrimental for DNA integrity in *Saccharomyces cerevisiae*. Genes Cells.

[34] Mizokami M, Orito E (1999). Molecular evolution of hepatitis viruses. Intervirol.

[35] Niu FX, He X, Huang YB, Liu JZ (2020). Biosensor-guided atmospheric and room-temperature plasma mutagenesis and shuffling for high-level production of shikimic acid from sucrose in *Escherichia coli*. J Agr Food Chem.

[36] Yu G, ClusterProfiler. An universal enrichment tool for functional and comparative study. CSH Lab. 2018;256784.

[37] Ferreira JA, Zwinderman AH (2006). On the Benjamini-Hochberg method. Ann Stat.

