# Supplementary material for: Key role of K+ and Ca2+ in high-yield ethanol production by S. Cerevisiae from concentrated sugarcane molasses
Source: Microb Cell Fact. 2024 May 9;23:123. doi: 10.1186/s12934-024-02401-5 (PMC11080136; doi:10.1186/s12934-024-02401-5)
Supplement: Supplementary file 2 — Supplementary Material 2 [file 12934_2024_2401_MOESM2_ESM.docx]

**Key effects of K^+^ and Ca^2+^ on *Saccharomyces cerevisiae*-mediated high-concentration ethanol fermentation using high-concentration sugarcane molasses**

**Wei-Yang Wang^1, 2^, Bei-Ping Wang^1^, Hai-Song Su^1^, Mei-Ming Wei^1^, Yu-Tuo Wei^2^ and Fu-Xing Niu^1^***

1 Guangxi Key Laboratory of Green Processing of Sugar Resources, Guangxi University of Science and Technology, Liuzhou, 545006, China.

2 Guangxi Microorganism and Enzyme Research Center of Engineering Technology, College of Life Science and Technology, Guangxi University, Nanning, Guangxi 530004, China

Email:

Wei-Yang Wang: 221055302@stdmail.gxust.edu.cn

Bei-Ping Wang: 221077197@stdmail.gxust.edu.cn

Hai-Song Su: 211058902@stdmail.gxust.edu.cn

Mei-Ming Wei: 211058960@stdmail.gxust.edu.cn

Yu-Tuo Wei: weiyutuo@gxu.edu.cn

Fu-Xing Niu: niufx3@gxust.edu.cn

*** Correspondence:**

Fu-Xing Niu: Guangxi Key Laboratory of Green Processing of Sugar Resources, Guangxi University of Science and Technology, Liuzhou, 545006, China. Email address: niufx3@gxust.edu.cn

## Supplement


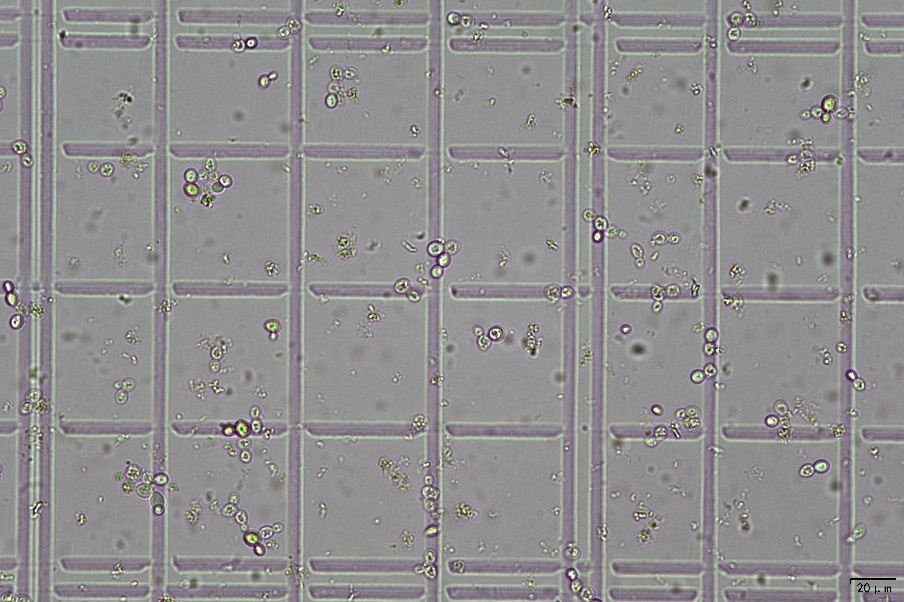


Supplementary Figure 1 *S. cerevisiae* GJ08 under 250 g/L sugarcane molasses.

As shown in the figure, the yeast cells show a state of aggregation and a large number of deaths.

## *S. cerevisiae* NGT-F1 screening

Supplementary Figure 2 ARTP mutation and adaptive evolution of high sucrose tolerance strains.

After ARTP mutagenesis, the mutant yeast cellsl library obtained was firstly cultured in YPD medium containing 300 g/L total sugar. After stable growth, the next round of mutagenesis was carried out again, and the total sugar in the medium was increased until to 400 g/L. The number of cells was counted by cell counting plates.


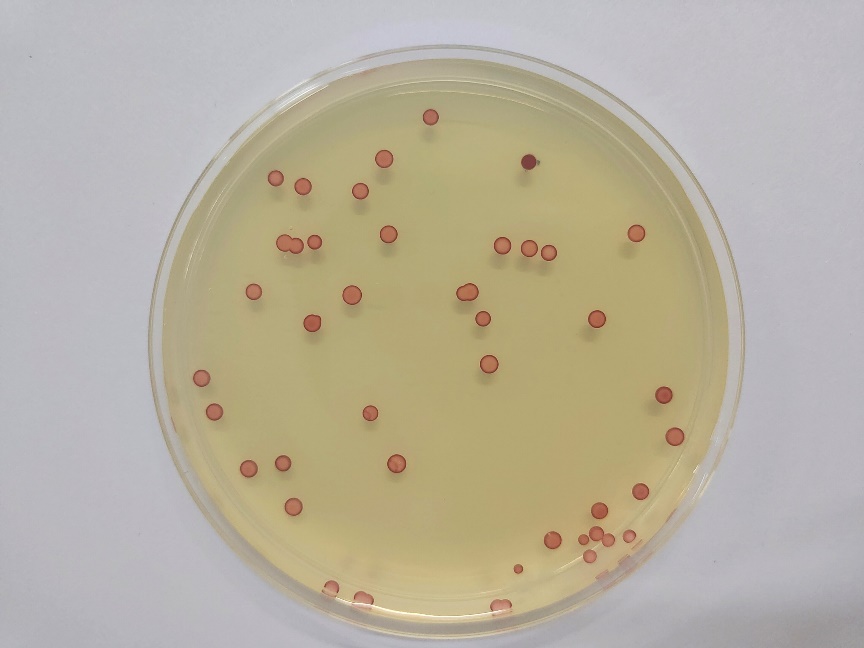


Supplementary Figure 3 TTC screening of high sucrose tolerance strains

20 mL Triphenyl-2H-tetrazoliumchloride (TTC) solution was introduced to react for 5 min, and the yeast strains with the earliest red and darkest color were selected

Supplementary Figure 4 Py-Fe3^+^ screening of high sucrose tolerance strains

Single *S. cerevisiae* colonies on the slant plates were transferred to 48-deep-well microtiter plates (DWMP) containing 1 mL specific medium, and cultivated under their specific environment. After the fermentation, DWMPs was left to rest for 30 min to allow *S. cerevisiae* strains to sink automatically. Then 120 µL of the fermentation supernatant (Five times dilution) was transferred to a 96-well enzyme label plate, and 80 µL 0.1M Fe(NO_3_)_3_was added for reaction at room temperature for 10 min. Then the absorbance was measured at OD_520_ nm.

Supplementary Figure 5 Ethanol synthesis yield rescreening of high sucrose tolerance strains (YPD medium containing 400 g/L total sugar)

Supplementary Figure 6 Optimization of sucrose ethanol synthesis by mutant strain. 300, 325, 350, 375, and 400 g/L total sugar were used to determine the conditions for optimal ethanol production.


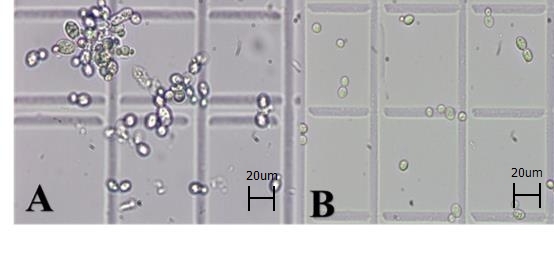


Supplementary Figure 7 Comparison of cell morphology between mutant yeast cells and non-mutant yeast cells at 400 g/L total sugar condition for 24 h.

(A) *S. cerevisiae* GJ08; (B) *S. cerevisiae* NGT-F1.

## *S. cerevisiae* NGW-F1 screening

Supplementary Figure 8 ARTP mutation and adaptive evolution of high temperature tolerance strains After ARTP mutagenesis, the mutant yeast cellsl library obtained was firstly cultured at 37℃. After stable growth, the next round of mutagenesis was carried out at 37℃. The number of cells was counted by cell counting plates.


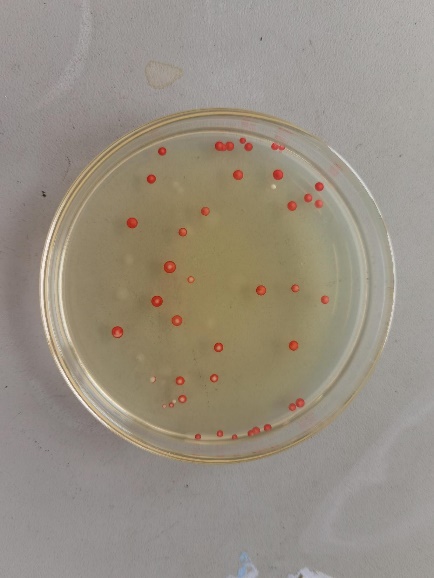


Supplementary Figure 9 TTC screening of high temperature tolerance strains

20 mL Triphenyl-2H-tetrazoliumchloride (TTC) solution was introduced to react for 5 min, and the yeast strains with the earliest red and darkest color were selected

Supplementary Figure 10 Py-Fe3^+^ screening of high temperature tolerance strains

Single *S. cerevisiae* colonies on the slant plates were transferred to 48-deep-well microtiter plates (DWMP) containing 1 mL specific medium, and cultivated under their specific environment. After the fermentation, DWMPs was left to rest for 30 min to allow *S. cerevisiae* strains to sink automatically. Then 120 µL of the fermentation supernatant (Five times dilution) was transferred to a 96-well enzyme label plate, and 80 µL 0.1M Fe(NO_3_)_3_was added for reaction at room temperature for 10 min. Then the absorbance was measured at OD_520_ nm.

Supplementary Figure 11 Ethanol synthesis yield rescreening of high temperature tolerance strains (37 ℃, YPD medium containing 400 g/L total sugar)


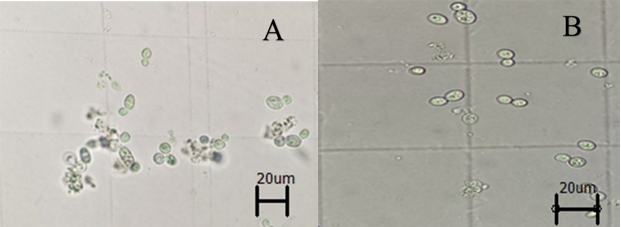


Supplementary Figure 12 Comparison of cell morphology between mutant yeast cells and non-mutant yeast cells at 37℃ for 24 h.

(A) *S. cerevisiae* GJ08; (B) *S. cerevisiae* NGW-F1.

## *S. cerevisiae* NGC-F1 screening

Supplementary Figure 13 ARTP mutation and adaptive evolution of high ethanol tolerance strains

After ARTP mutagenesis, the mutant library was placed in YPD medium containing 10% (v/v) ethanol for subculture. After 3 generations, all yeast cells were collected for the second ARTP mutagenesis until the number of cells reached the temperature state.


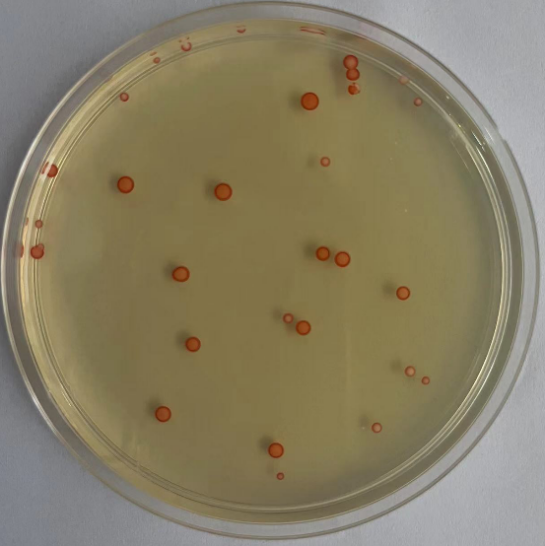


Supplementary Figure 14 TTC screening of high ethanol tolerance strains

20 mL Triphenyl-2H-tetrazoliumchloride (TTC) solution was introduced to react for 5 min, and the yeast strains with the earliest red and darkest color were selected

Supplementary Figure 15 Py-Fe3^+^ screening of high ethanol tolerance strains

Single *S. cerevisiae* colonies on the slant plates were transferred to 48-deep-well microtiter plates (DWMP) containing 1 mL specific medium, and cultivated under their specific environment. After the fermentation, DWMPs was left to rest for 30 min to allow *S. cerevisiae* strains to sink automatically. Then 120 µL of the fermentation supernatant (Five times dilution) was transferred to a 96-well enzyme label plate, and 80 µL 0.1M Fe(NO_3_)_3_was added for reaction at room temperature for 10 min. Then the absorbance was measured at OD_520_ nm.

Supplementary Figure 16 Ethanol synthesis yield rescreening of high ethanol tolerance strains (YPD medium containing 250 g/L total sugar)


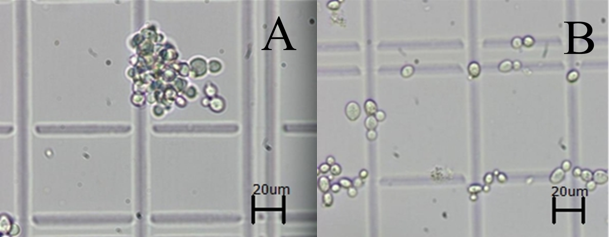


Supplementary Figure 17 Comparison of cell morphology between mutant yeast cells and non-mutant yeast cells at 10% (v/v) ethanol for 24 h.

(A) *S. cerevisiae* GJ08; (B) *S. cerevisiae* NGC-F1.

## *S. cerevisiae* NGK^+^-F1 screening

Supplementary Figure 18 Effect of different concentrations of K^+^ on the growth of *S.cerevisiae*

Supplementary Figure 19 ARTP mutation and adaptive evolutionary breeding in high concentration K^+^. After ARTP mutagenesis, the mutant yeast cellsl library obtained was firstly cultured in YPD medium containing 8 g/L K^+^. After stable growth, the next round of mutagenesis was carried out in YPD medium containing 12 g/L K^+^, and to YPD medium containing 16 g/L K^+^. The number of cells was counted by cell counting plates.


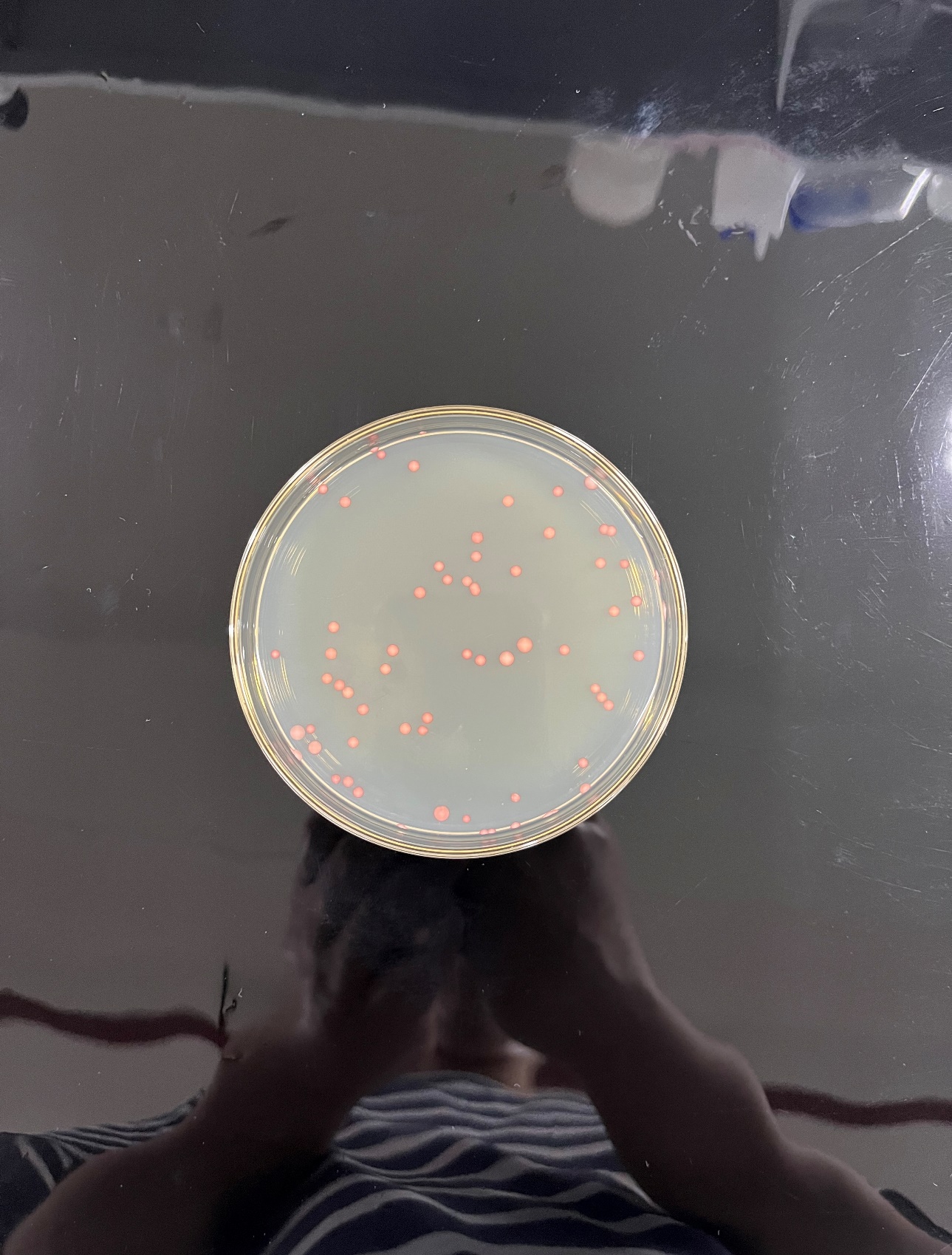


Supplementary Figure 20 TTC screening of high K^+^ tolerance strains

20 mL Triphenyl-2H-tetrazoliumchloride (TTC) solution was introduced to react for 5 min, and the yeast strains with the earliest red and darkest color were selected

Supplementary Figure 21 Py-Fe3^+^ screening of high K^+^ tolerance strains

Single *S. cerevisiae* colonies on the slant plates were transferred to 48-deep-well microtiter plates (DWMP) containing 1 mL specific medium, and cultivated under their specific environment. After the fermentation, DWMPs was left to rest for 30 min to allow *S. cerevisiae* strains to sink automatically. Then 120 µL of the fermentation supernatant (Five times dilution) was transferred to a 96-well enzyme label plate, and 80 µL 0.1M Fe(NO_3_)_3_ was added for reaction at room temperature for 10 min. Then the absorbance was measured at OD_520_ nm.

Supplementary Figure 22 Ethanol synthesis yield rescreening of high K^+^ (16 g/L)tolerance strains


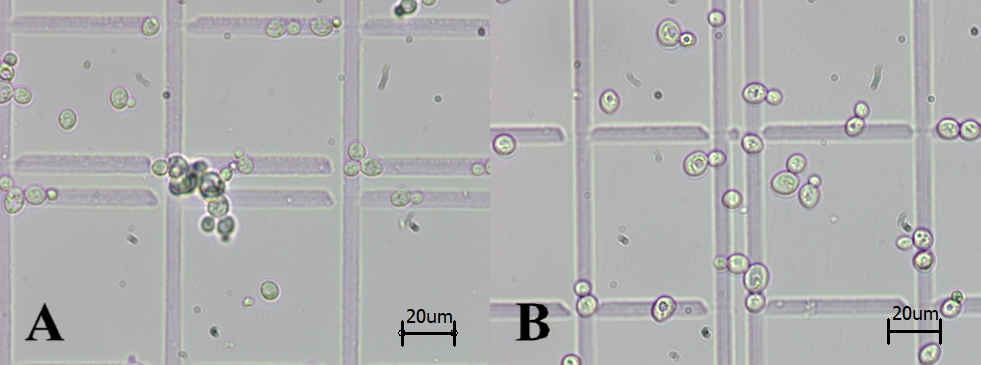


Supplementary Figure 23 Comparison of cell morphology between mutant yeast cells and non-mutant yeast cells at 16 g/L K^+^ for 24 h.

(A) *S. cerevisiae* GJ08; (B) *S. cerevisiae* NGK^+^-F1.

## *S. cerevisiae* NGCa^2+^-F1 screening

Supplementary Figure 24 Effect of different concentrations of Ca^2+^ on the growth of *S.cerevisiae*

Supplementary Figure 25 ARTP mutation and adaptive evolutionary breeding in high concentration Ca^2+^. After ARTP mutagenesis, the mutant yeast cellsl library obtained was firstly cultured in YPD medium containing 4 g/L Ca^2+^. After stable growth, the next round of mutagenesis was carried out in YPD medium containing 6 g/L Ca^2+^, and to YPD medium containing 8 g/L Ca^2+^(Two mutagenesis was performed at 8 g/L). The number of cells was counted by cell counting plates.


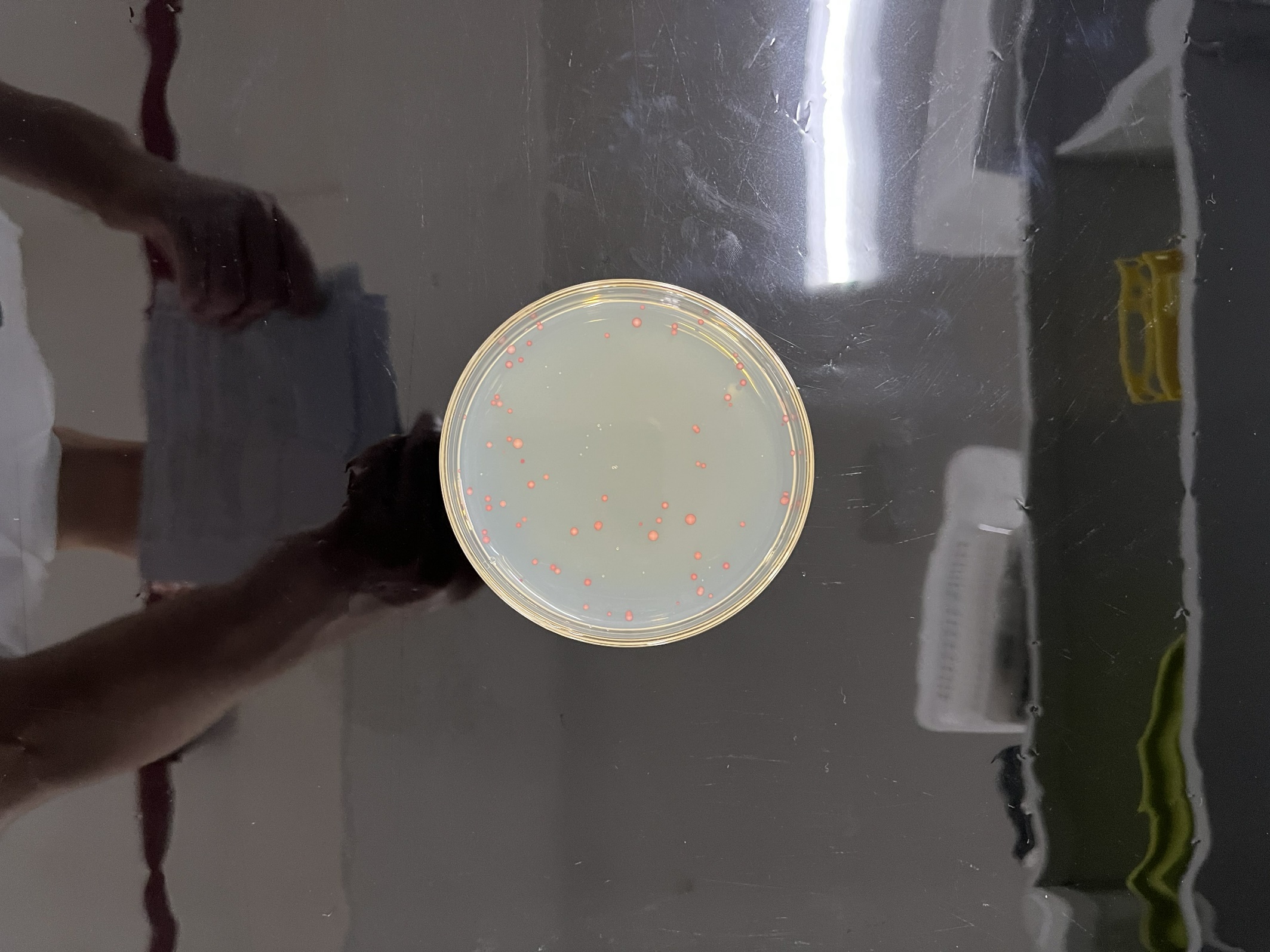


Supplementary Figure 26 TTC screening of high Ca^+^ tolerance strains

20 mL Triphenyl-2H-tetrazoliumchloride (TTC) solution was introduced to react for 5 min, and the yeast strains with the earliest red and darkest color were selected

Supplementary Figure 27 Py-Fe3^+^ screening of high Ca^2+^ tolerance strains.

Single *S. cerevisiae* colonies on the slant plates were transferred to 48-deep-well microtiter plates (DWMP) containing 1 mL specific medium, and cultivated under their specific environment. After the fermentation, DWMPs was left to rest for 30 min to allow *S. cerevisiae* strains to sink automatically. Then 120 µL of the fermentation supernatant (Five times dilution) was transferred to a 96-well enzyme label plate, and 80 µL 0.1M Fe(NO_3_)_3_was added for reaction at room temperature for 10 min. Then the absorbance was measured at OD_520_ nm.

Supplementary Figure 28 Ethanol synthesis yield rescreening of high Ca^2+^ (8 g/L)tolerance strains


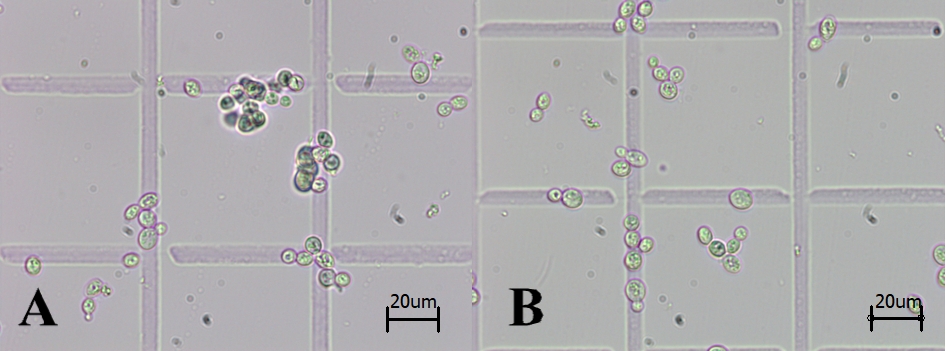


Supplementary Figure 29 Comparison of cell morphology between mutant yeast cells and non-mutant yeast cells at 8 g/L Ca^2+^ for 24 h.

(A) *S. cerevisiae* GJ08; (B) *S. cerevisiae* NGCa^2+^-F1.

## *S. cerevisiae* NGK^+^&Ca^2+^-F1 screening

Supplementary Figure 30 Effect of different concentrations of K^+^＆Ca^2+^ on the growth of *S.cerevisiae*

Supplementary Figure 31 ARTP mutation and adaptive evolutionary breeding in high concentration K^+^&Ca^2+^ environments. After ARTP mutagenesis, the mutant yeast cells library obtained was firstly cultured in YPD medium containing (8 g/L ^+^4 g/L) K^+^&Ca^2+^. After stable growth, the next round of mutagenesis was carried out in YPD medium containing (12 g/L ^+^6 g/L) K^+^&Ca^2+^, and to YPD medium containing (16 g/L ^+^8 g/L) K^+^&Ca^2+^ (Two mutagenesis was performed at (16 g/L ^+^8 g/L) K^+^&Ca^2+^). The number of cells was counted by cell counting plates.


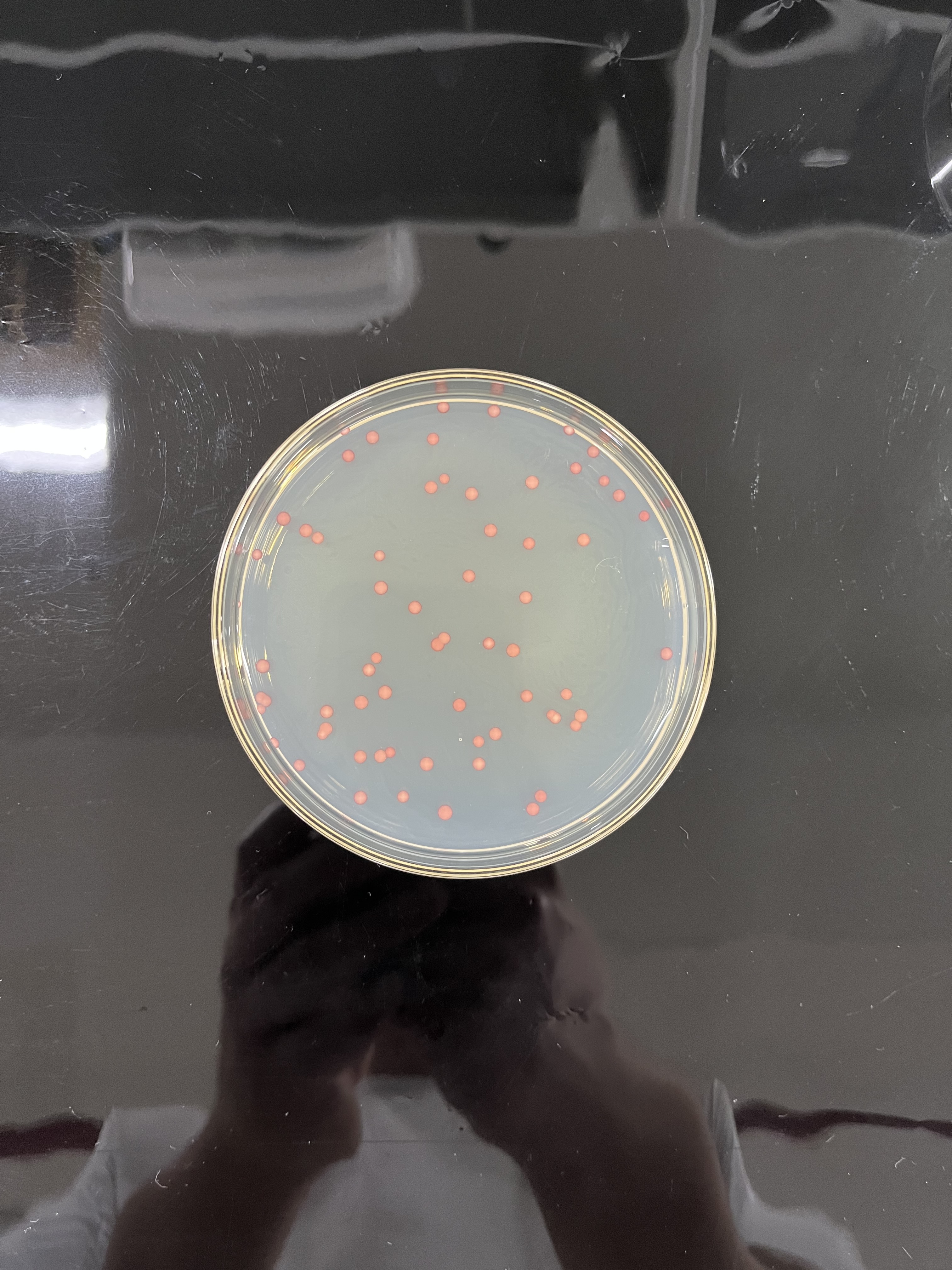


Supplementary Figure 32 TTC screening of high K^+^＆Ca^2+^ tolerance strains

20 mL Triphenyl-2H-tetrazoliumchloride (TTC) solution was introduced to react for 5 min, and the yeast strains with the earliest red and darkest color were selected

Supplementary Figure 33 Py-Fe3^+^ screening of high K^+^&Ca^2+^ tolerance strains.

Single *S. cerevisiae* colonies on the slant plates were transferred to 48-deep-well microtiter plates (DWMP) containing 1 mL specific medium, and cultivated under their specific environment. After the fermentation, DWMPs was left to rest for 30 min to allow *S. cerevisiae* strains to sink automatically. Then 120 µL of the fermentation supernatant (Five times dilution) was transferred to a 96-well enzyme label plate, and 80 µL 0.1M Fe(NO_3_)_3_was added for reaction at room temperature for 10 min. Then the absorbance was measured at OD_520_ nm.

Supplementary Figure 34 Ethanol synthesis yield rescreening of high K^+^&Ca^2+^ (16 g/L ^+^8 g/L) tolerance strains


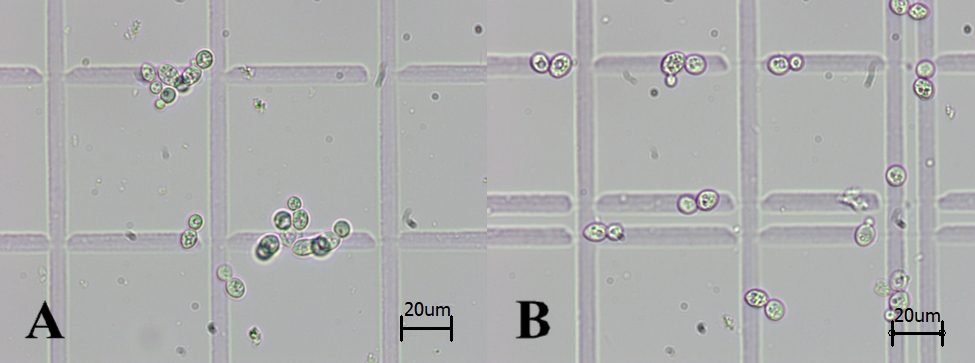


Supplementary Figure 35 Comparison of cell morphology between mutant yeast cells and non-mutant yeast cells at (16 g/L +8 g/L) K^+^&Ca^2+^ for 24 h.

(A) *S. cerevisiae* GJ08; (B) *S. cerevisiae* NGK^+^&Ca^2+^-F1.

## *S. cerevisiae* NGTM-F1 screening

Supplementary Figure 36 Effects of sugarcane molasses with different sugar concentrations on the growth of *S.cerevisiae*

Supplementary Figure 37 ARTP mutation and adaptive evolutionary breeding in high concentration sugarcane molasses. After ARTP mutagenesis, the mutant yeast cellsl library obtained was firstly cultured in YPD medium containing 250 g/L total sugar. After stable growth, the next round of mutagenesis was carried out in YPD medium containing 270 g/L total sugar, and finally to YPD medium containing 300 g/L total sugar (Two mutagenesis was performed at 300 g/L total sugar). The number of cells was counted by cell counting plates.


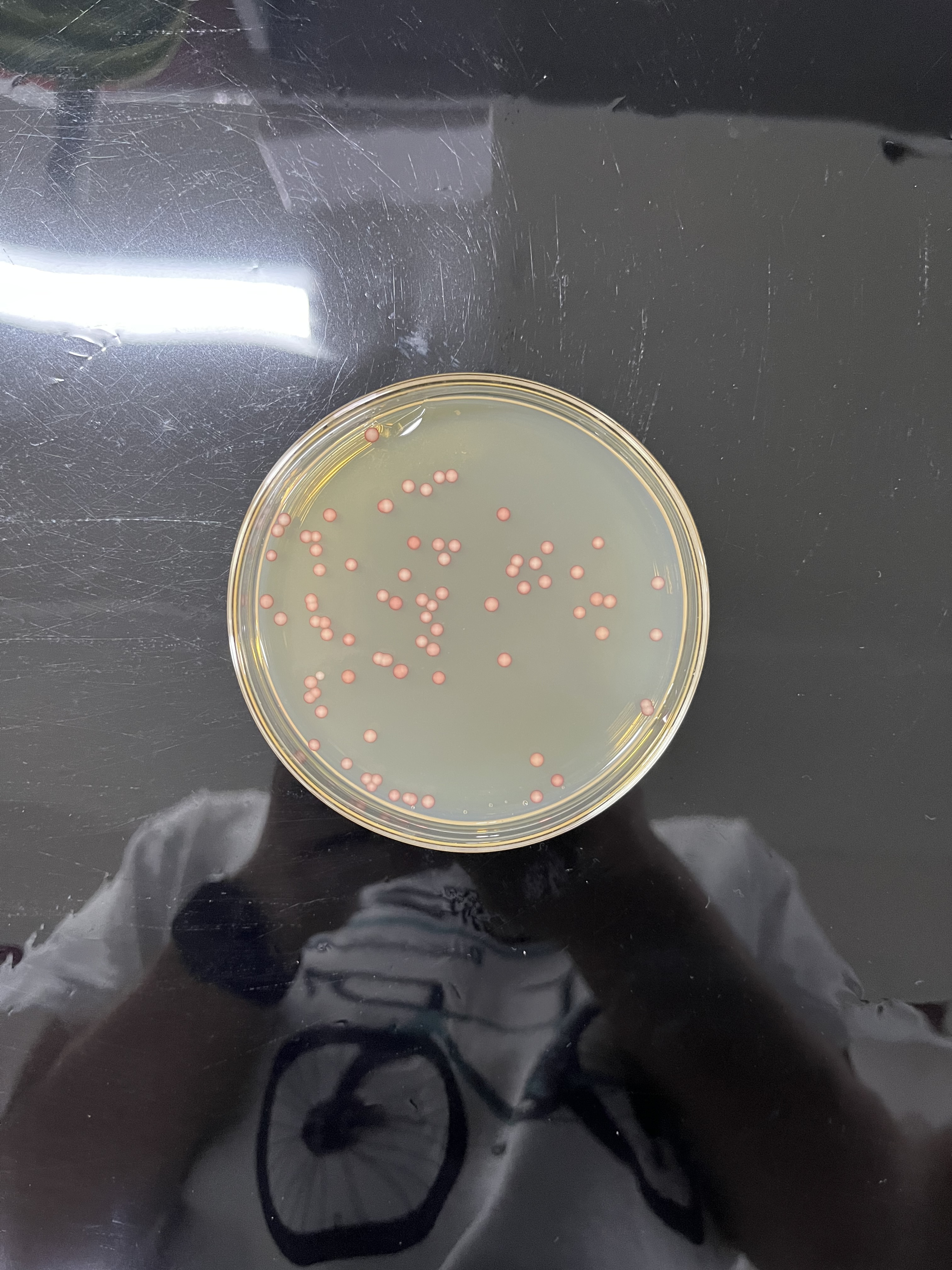


Supplementary Figure 38 TTC screening of high sugarcane molasses tolerance strains

20 mL Triphenyl-2H-tetrazoliumchloride (TTC) solution was introduced to react for 5 min, and the yeast strains with the earliest red and darkest color were selected

Supplementary Figure 39 Py-Fe3^+^ screening of high sugarcane molasses tolerance strains.

Single *S. cerevisiae* colonies on the slant plates were transferred to 48-deep-well microtiter plates (DWMP) containing 1 mL specific medium, and cultivated under their specific environment. After the fermentation, DWMPs was left to rest for 30 min to allow *S. cerevisiae* strains to sink automatically. Then 120 µL of the fermentation supernatant (Five times dilution) was transferred to a 96-well enzyme label plate, and 80 µL 0.1M Fe(NO_3_)_3_was added for reaction at room temperature for 10 min. Then the absorbance was measured at OD_520_ nm.

Supplementary Figure 40 Ethanol synthesis yield rescreening of high sugarcane molasses (250 g/L total sugar) tolerance strains


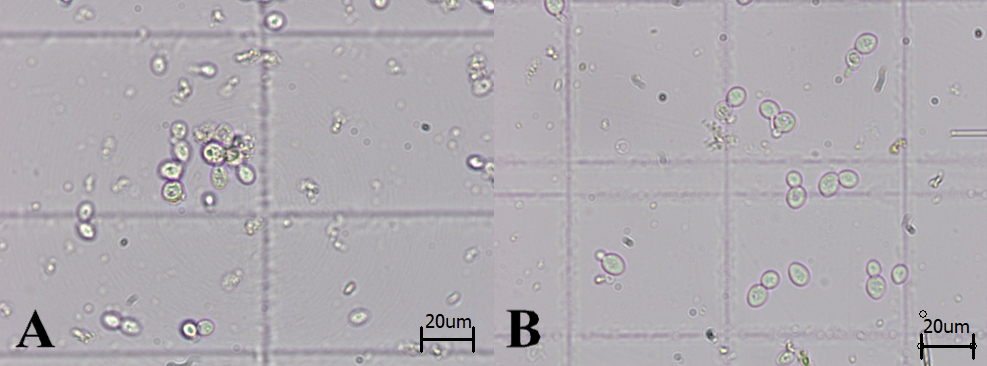


Supplementary Figure 41 Comparison of cell morphology between mutant yeast cells and non-mutant yeast cells at high sugarcane molasses (300 g/L total sugar) for 24 h.

(A) *S. cerevisiae* GJ08; (B) *S. cerevisiae* NGTM-F1.

## Optimization of fermentation conditions

Supplementary Figure 42 Optimization of different inoculations.

A: Ethanol synthesis under different inoculations; B: Residual sugar content under different inoculations.

Supplementary Figure 43 Optimization of different urea additions.

A: Ethanol synthesis under different urea additions; B: Residual sugar content under different urea additions.

## Whole genome resequencing GO enrich analysis

Supplementary Figure 44

Whole genome resequencing GO enrich analysis. A: *S. cerevisiae* NGCa^2+^-F1;

B: *S. cerevisiae* NGK^+^-F1.

## Comparative transcriptomic GO Term analysis

Supplementary Figure 45

Comparative transcriptomic GO Term analysis. A: *S. cerevisiae* NGCa^2+^-F1 compared with *S. cerevisiae* GJ08 under 8 g/L Ca^2+^; B: *S. cerevisiae* NGK^+^-F1 compared with *S. cerevisiae* GJ08 under 16 g/L K^+^.

Supplementary Table 1 Statistics of whole genome resequencing

| Sample | Genome Bases | Covered Bases | ^*1^Coverage (%) | Clean Bases (bp) | ^*2^Ratio of Bases (%) |
| --- | --- | --- | --- | --- | --- |
| *S. cerevisiae* NGK^+^-F1 | 12157105 | 12078089 | 99.35 | 2349391112 | 99.31 |
| *S. cerevisiae* NGCa^2+^-F1 | 12157105 | 12081825 | 99.38 | 2761554130 | 99.19 |
| *S. cerevisiae* NGK^+^&Ca^2+^-F1 | 12157105 | 12078992 | 99.36 | 2461507294 | 99.14 |
| *S. cerevisiae* NGTM-F1 | 12157105 | 12082816 | 99.39 | 4081672376 | 98.97 |

^*1^ Covered Bases/ Genome Bases*100%

^*2^ Clean Bases as a percentage of the original sequenced bases
